# Leptospiral dissemination is restrained by liver macrophages through Clec4d-driven capture via C/EBPβ activation

**DOI:** 10.1371/journal.ppat.1014232

**Published:** 2026-05-13

**Authors:** Xi Chen, Xin Liu, Jiuxi Liu, Haixin Huang, Wenlong Zhang, Xufeng Xie, Yongguo Cao

**Affiliations:** 1 Key Laboratory for Zoonosis Research, Ministry of Education, College of Veterinary Medicine, Jilin University, Changchun, People’s Republic of China; 2 Department of Clinical Veterinary Medicine, College of Veterinary Medicine, Jilin University, Changchun, People’s Republic of China; Medical College of Wisconsin, UNITED STATES OF AMERICA

## Abstract

Leptospirosis, caused by pathogenic *Leptospira* species, is a globally significant zoonotic disease with high morbidity and mortality. However, the organs or cells mainly involved in capturing circulating leptospires and the related mechanisms remain poorly understood. In this study, we firstly proved that the liver was the primary organ that captured leptospires during the very early intravascular phase of infection in mice. Then, we used unbiased flow sorting of leptospires-positive cells and intravital microscopy of mice infected with leptospires, and found that liver macrophages were the main leptospires-capturing immune cells. The depletion of liver macrophages abolished the ability of liver to capture leptospires and prompted leptospiral spread in other organs. The C-type lectin receptor signaling pathway and *Clec4d* were identified as the differential pathways and gene through RNA-seq analysis, respectively. The ectopic expression of Clec4d in HEK-293T cells or treatment with a Clec4d inhibitor, mannan proved that Clec4d functioned as a capture receptor of leptospires. Mechanistically, the transcription factor CCAAT/enhancer-binding protein beta (C/EBPβ) was activated and directly bound to the promoter of *Clec4d* to promote the expression of Clec4d in liver macrophages, thereby enhancing leptospiral capture. Mice treated with C/EBPβ inhibitor showed a significant inhibition of liver macrophages in capturing leptospires and increased leptospiral load in other organs. Our findings identify a novel mechanism by which the liver macrophages restrict leptospiral dissemination through C/EBPβ-Clec4d axis, and suggest a therapeutic strategy to prevent leptospiral dissemination through enhancing liver macrophages functions.

## Author summary

Leptospirosis, a globally significant zoonotic disease caused by pathogenic *Leptospira* spp, poses a major threat to human health due to its high morbidity and mortality. However, how circulating leptospires are captured and restricted in the body remains unclear. In this study, we found that the liver acted as a crucial immune barrier during the very early intravascular phase of infection, with liver-resident macrophages (Kupffer cells, KCs) being the key cells that captured leptospires from the bloodstream. We identified a C-type lectin receptor, Clec4d, as the specific receptor mediating leptospiral capture by KCs. Mechanistically, the transcription factor C/EBPβ directly drives *Clec4d* expression in KCs, and disrupting this C/EBPβ-Clec4d axis impairs leptospiral capture, leading to leptospires dissemination. Our work uncovers a novel liver-centric immune mechanism against leptospirosis and suggests that targeting the C/EBPβ-Clec4d axis to enhance Kupffer cell function could be a promising strategy to prevent leptospiral spread and improve disease outcomes.

## Introduction

Leptospirosis is a zoonotic bacterial disease caused by pathogenic spirochetes of the genus *Leptospira*, which occurs across all continents [1,2]. While infection may be asymptomatic, symptomatic cases can range from mild to severe manifestations. Severe illness is marked by jaundice and/or multiple organ failure, and may lead to death [3]. Globally, an estimated one million leptospirosis cases occur annually, resulting in approximately 60,000 fatalities [3]. *Leptospira* that invade the body rapidly disseminate through the bloodstream to the entire system and ultimately colonize the kidneys [1,4,5]. Previous studies have indicated that the degree of control achieved during the early stages of leptospiral infection determines the extent of subsequent renal colonization by the pathogen [6]. However, the specific host immune mechanisms that mediate effective early control of leptospiral dissemination and limit renal colonization remain incompletely elucidated, highlighting the need for further investigation into the very early intravascular phase of infection.

The host innate immune system, particularly neutrophils and macrophages, constitutes the primary line of defense against leptospiral invasion [7]. Early *in vitro* and *ex vivo* studies have uncovered distinct roles for these two cell types in leptospiral infection. While neutrophils readily bind to pathogenic leptospires on their cell surface, they rarely initiate effective phagocytosis, suggesting a limited capacity for direct bacterial clearance [8]. By contrast, macrophages exhibit robust anti-leptospiral activity *in vitro*, where they not only phagocytose and intracellularly eliminate leptospires but also secrete pro-inflammatory cytokines to orchestrate systemic immune responses [7,9]. Macrophage surface pattern recognition receptors (PRRs), including Toll-like receptors (TLR2, TLR4) and complement receptors, further enable pathogen recognition and antigen presentation, bridging innate and adaptive immunity to mount sustained anti-leptospiral defenses [9]. While these *in vitro* monoculture systems and isolated cell assays have greatly facilitated our understanding of leptospiral-host cell interactions, the specific mechanisms governing these processes within the host’s complex, tissue-specific *in vivo* microenvironments remain largely undefined.

KCs have been widely documented to mediate the sequestration of a broad spectrum of circulating pathogens, including fungal pathogens such as *Cryptococcus neoformans* and *Candida albicans* [10,11]. Beyond KCs, liver endothelial cells have also been demonstrated to perform a specialized role in trapping viruses and bacteriophages within the circulating blood [12–14]. In addition, splenic red pulp macrophages play a critical role in clearing blood-borne encapsulated bacteria (e.g., *Streptococcus pneumoniae*, *Haemophilus influenzae*, and *Neisseria meningitidis*) to thwart systemic invasive infections [15–17]. However, it remains to be determined which organ serves as the primary site for leptospiral sequestration, and which cell populations within these organs act as the dominant effectors of leptospire capture. Furthermore, the molecular mechanisms underlying this process remain to be fully elucidated.

In this study, we discovered that the liver, compared to the kidneys, spleen, and lungs, captured a significantly higher leptospiral load in the early phase post-infection, and that this phenotype was independent of leptospiral strain and host sex. Through a combination of *in vivo* and *in vitro* approaches, we identified KCs, rather than neutrophils, as the principal immune cells responsible for this rapid liver capture of leptospires. Mechanistically, this recognition was dependent on the C-type lectin receptor Clec4d. Furthermore, we elucidated that following infection, the transcription factor C/EBPβ was activated and orchestrated the upregulation of Clec4d, thereby potentiating the phagocytic capacity of macrophages against *Leptospira*. This comprehensive understanding highlights the significance of hepatic macrophage function in the host response to *Leptospira* infection and establishes a foundational mechanism for developing targeted, host-directed therapeutic strategies for enhancing liver macrophage microbicidal activity to prevent systemic leptospiral spread.

## Results

### Liver is the primary organ for capturing leptospires during the very early intravascular phase of infection

**Fig 1:**
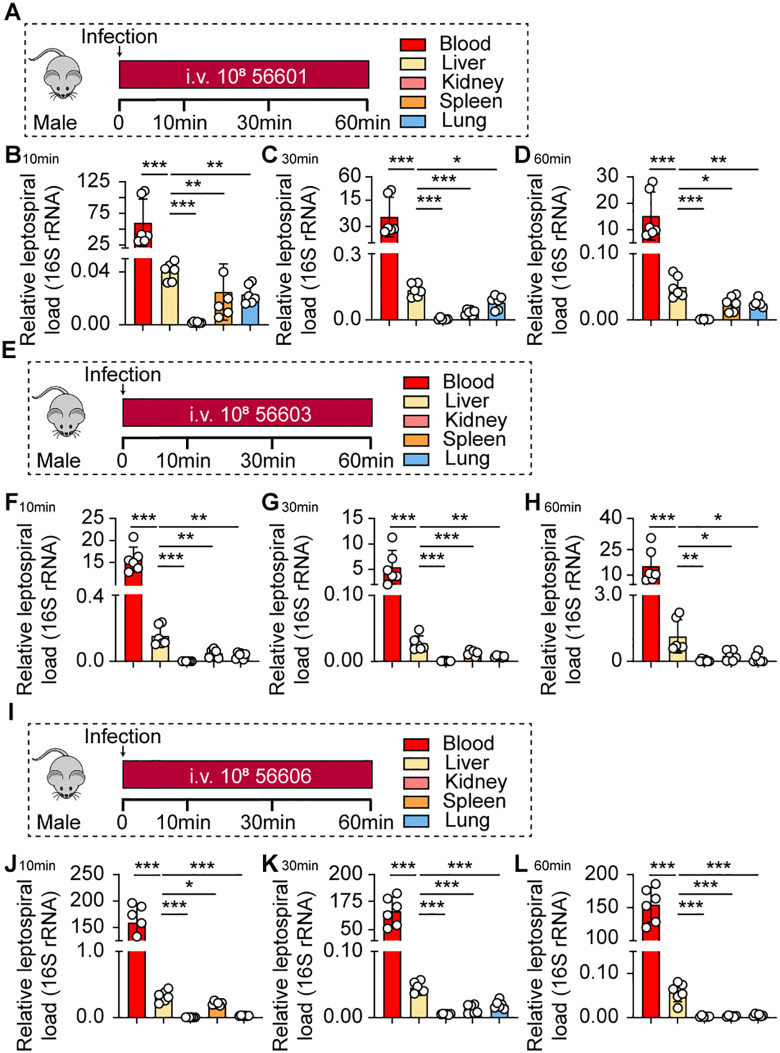
The liver is the primary organ for capturing leptospires during the early phase of infection. **(A)** Experimental schematic: Male C57BL/6 mice were intravenously infected with 10⁸ pathogenic *L. interrogans* (strains 56601). Leptospiral load in blood, liver, kidneys, spleen, and lungs was quantified by qPCR at 10, 30, and 60 min post-infection (p.i.). **(B-D)** Leptospiral load in various organs at 10 min **(B)**, 30 min **(C)**, and 60 min (D) after infection with strain 56601 (n = 6). **(E)** Experimental schematic: Male C57BL/6 mice were intravenously infected with 10⁸ pathogenic *L. interrogans* (strains 56603). Leptospiral load in blood, liver, kidneys, spleen, and lungs was quantified by qPCR at 10, 30, and 60 min post-infection (p.i.). **(F-H)** Leptospiral load in various organs at 10 min **(F)**, 30 min **(G)**, and 60 min (H) after infection with strain 56603 (n = 6). **(I)** Experimental schematic: Male C57BL/6 mice were intravenously infected with 10⁸ pathogenic *L. interrogans* (strains 56606). Leptospiral load in blood, liver, kidneys, spleen, and lungs was quantified by qPCR at 10, 30, and 60 min post-infection (p.i.). **(J-L)** Leptospiral load in various organs at 10 min **(J)**, 30 min **(K)**, and 60 min (L) after infection with strain 56606 (n = 6). Data are presented as mean ± SEM. Statistical significance was determined with Student’s t test (unpaired, two-tailed). *p < 0.05, **p < 0.01, ***p < 0.001.

To investigate the capture of leptospires by various organs during the early stage of infection, C57BL/6 male mice were intravenously infected with *Leptospira interrogans* serovar Lai strain Lai (56601) (Fig 1A), a strain identified as one of the primary pathogenic isolates responsible for severe leptospirosis in human and animal populations across China [18]. The leptospiral loads in the blood, liver, kidneys, spleen, and lungs were quantified at 10, 30, and 60 minutes post-infection via qPCR. The results demonstrated that the liver harbored a significantly higher leptospiral load compared to the kidneys, spleen, and lungs (Fig 1B–1D). To ensure the robustness and generalizability of this finding, we repeated the infection experiment using two distinct *L. interrogans* strains (56603 and 56606) (Fig 1E and 1I), and the leptospiral load in the corresponding organs at the same time points was quantified. Consistent with our initial findings, livers infected with either strain 56603 (Fig 1F–1H) or strain 56606 (Fig 1J–1L) exhibited the highest leptospiral loads. Studies have reported that sex impacts severity outcomes of leptospirosis [19–22]. To control for potential sex-based differences, female mice were infected with strain 56601 (S1A Fig), and the organ-specific leptospiral load was determined at the aforementioned time points. Consistent with the findings in males, the liver leptospiral load was significantly higher than that in other organs (S1B–S1D Fig). Although the liver was the predominant organ for leptospiral capture in both sexes, we observed that at 10 min post‑infection the hepatic bacterial load was significantly higher in female mice than in male mice. This may be due to the difference in susceptibility to leptospirosis between male and female mice. To rule out potential confounding effects from blood residual in the tissues, cardiac perfusion was performed on mice prior to tissue collection, and the leptospiral load in the perfused organs was further quantified (S2A Fig). Even after this procedure, the liver remained the organ with the most abundant leptospiral colonization (S2B–S2D Fig).

Given that *Leptospira* naturally invade the host through mucosal surfaces or damaged skin rather than via intravenous injection, we further investigated whether the route of inoculation influences the early organ distribution pattern. To this end, C57BL/6 male mice were intraperitoneally infected with strain 56601, and the leptospiral loads in the same set of organs were quantified at 10, 30, and 60 minutes post-infection. Notably, the liver again exhibited the highest leptospiral burden among all organs tested, mirroring the results observed following intravenous infection (S4A–S4C Fig). Consistent findings were also observed in female mice following intraperitoneal infection (S4D–S4F Fig). Furthermore, to exclude the potential impact of blood residual, male mice were subjected to cardiac perfusion prior to tissue collection after intraperitoneal infection, and the leptospiral load in the perfused organs was quantified. Under these conditions, the liver remained the predominant organ for leptospiral capture (S4G–S4I Fig). These results collectively indicate that the preferential capture of leptospires by the liver during the very early intravascular phase of infection is independent of the inoculation route. These data collectively indicated that the liver served as the predominant organ for capturing leptospires during the very early intravascular phase of infection.

### Liver macrophages are the primary immune cells responsible for capturing leptospires

**Fig 2:**
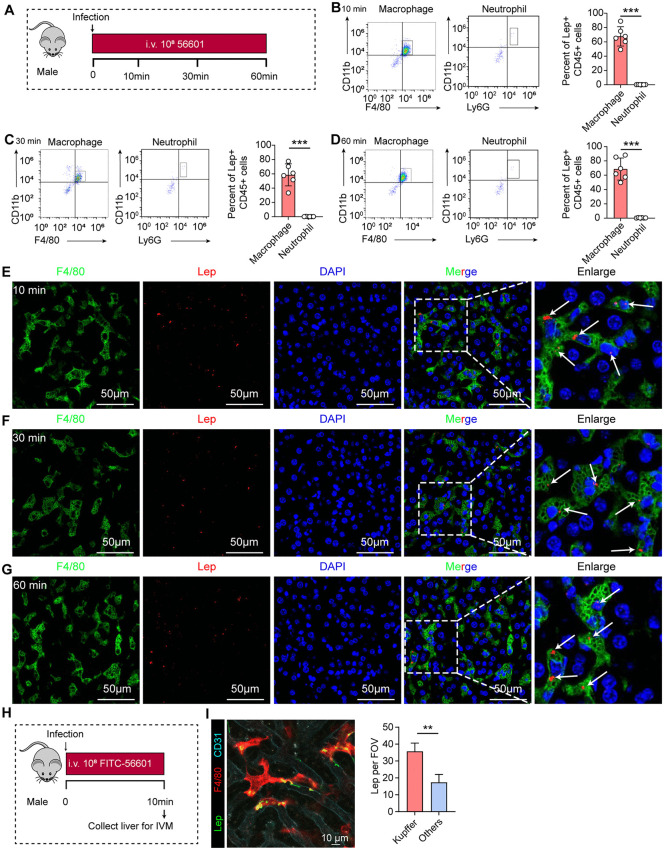
Liver macrophages are the primary cells responsible for the rapid capture of leptospires. **(A)** Schematic of the experimental workflow for flow cytometry and IF analysis. Male C57BL/6 mice were intravenously infected with 10⁸ FITC-labeled leptospires. Livers were collected at 10, 30, and 60 min post-infection (p.i.) for the isolation of liver non-parenchymal cells (LNPCs) for flow cytometric analysis and for tissue sectioning for IF staining. **(B-D)** Flow cytometric analysis of phagocytic cells in the liver at 10 min **(B)**, 30 min **(C)**, and 60 min (D) p.i. For each time point, the left panel shows a representative dot plot of FITC+ macrophages (F4/80+ CD11b+), the middle panel shows a representative dot plot of FITC+ neutrophils (Ly6G+ F4/80- CD11b+), and the right panel shows the quantitative comparison of the number of leptospires-positive macrophages and neutrophils (n = 5). **(E-G)** Representative IF images of liver sections at 10 min **(E)**, 30 min **(F)**, and 60 min (G) p.i. Macrophages are stained with anti-F4/80 (green), leptospires are stained with a specific antibody (red), and nuclei are counterstained with DAPI (blue). Scale bars, 50 µm. **(H)** Schematic of the intravital microscopy (IVM) experimental setup. Mice were intravenously infected with 10⁸ FITC-labeled leptospires and the liver was imaged at 10 min p.i. **(I)** A representative still frame from the IVM video. Leptospires (green) are shown in close association with KCs (stained with anti-F4/80 in red) within the liver sinusoids. Endothelial cells are shown in blue. KC-associated leptospires (Lep) per ﬁeld of view (FOV) are presented as mean ± SEM 3 images. Scale bar, 10 µm. Data are presented as mean ± SEM. Statistical significance was determined with Student’s t test (unpaired, two-tailed). ***p < 0.001.

To perform an unbiased test of which type of immune cells in the liver capture leptospires during the very early intravascular phase (10–60 min post-infection), we infected mice with FITC-labeled leptospires and sacrificed them at 10, 30, and 60 minutes post-infection (p.i.). Livers were harvested, and liver leukocytes (non-parenchymal cells, LNPCs) were isolated for analysis by flow cytometry (Fig 2A). FITC+ cells were defined as those that had captured *Leptospira* (S3 Fig). Flow cytometric analysis showed that at all time points examined (10, 30, and 60 minutes p.i.), the vast majority of FITC+ cells were found within the F4/80+ CD11b+ cell compartment (Fig 2B–2D). In contrast, neutrophils (Ly6G+ CD11b+) contained significantly fewer leptospires at these early time points (Fig 2B–2D). These results demonstrate that KCs are the predominant phagocytic cells responsible for the rapid capture of leptospires from the circulation within the first hour of systemic infection.

To further validate the conclusion that liver macrophages are the primary cells acquiring leptospires at early time points, we performed immunofluorescence (IF) staining on liver sections from mice infected for 10, 30, and 60 minutes (Fig 2A). Leptospires were specifically labeled using a polyclonal antibody, and macrophages were identified by staining for the F4/80 marker. IF microscopy analysis revealed extensive co-localization of leptospires with F4/80+ cells, demonstrating that leptospires were efficiently captured by macrophages within the liver tissue (Fig 2E–2G). These histological observations provide direct visual evidence that KCs act as the predominant capture cells during the early phase of systemic leptospiral infection.

To further confirm the leptospire-capturing macrophages in the livers of mice, we visualized the behavior of leptospires in liver sinusoids by time-lapse intravital microscopy (IVM) imaging (Fig 2H). In the first 10 minutes upon intravenous inoculation, migrating leptospires in liver sinusoids of mice were predominantly immobilized near KCs (Fig 2I and S1 Movie). IVM data showed that leptospires capture in the liver is predominantly mediated by KCs.

**Fig 3:**
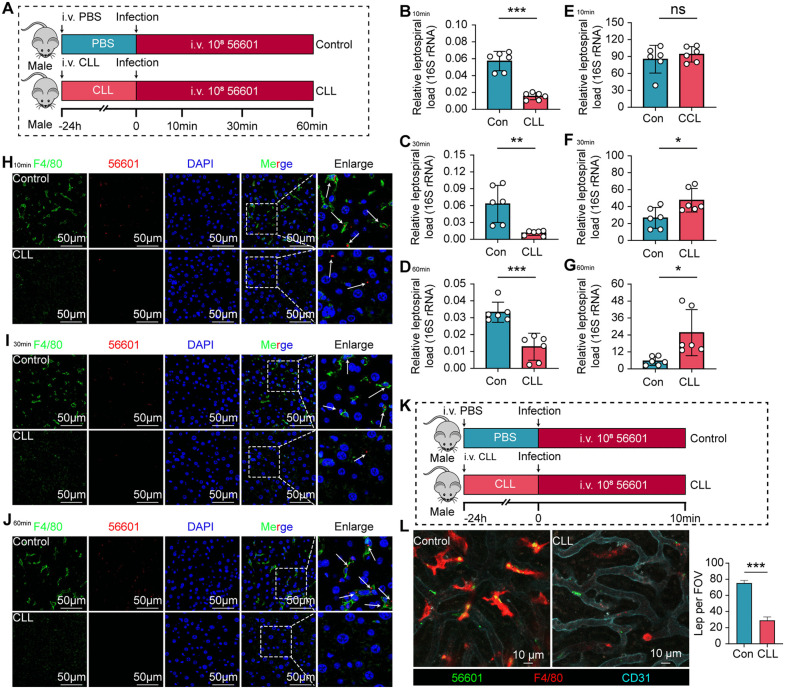
Depletion of liver macrophages impairs the early capture of leptospires. **(A)** Schematic of the experimental design for liver macrophage depletion and leptospiral infection. Mice were intravenously administered clodronate liposomes (CLL) or PBS 24 hours prior to infection with 10⁸ leptospires. Leptospiral load in the liver was quantified by qPCR, and liver sections were analyzed by IF at 10, 30, and 60 min post-infection (p.i.). **(B-D)** Liver leptospiral load in control and macrophage-depleted (CLL) mice at 10 min **(B)**, 30 min **(C)**, and 60 min (D) p.i. (n = 6). **(E-G)** Blood leptospiral load in control and macrophage-depleted (CLL) mice at 10 min **(E)**, 30 min **(F)**, and 60 min (G) p.i. (n = 6). **(H-J)** Representative IF images of liver sections from control (upper panels) and CLL-treated (lower panels) mice at 10 min **(H)**, 30 min **(I)**, and 60 min (J) p.i. Macrophages are stained with anti-F4/80 (green), leptospires with a specific antibody (red), and nuclei with DAPI (blue). Scale bars, 50 µm. Arrows indicate leptospires colocalized with macrophages. **(K)** Schematic of the intravital microscopy (IVM) experimental setup in macrophage-depleted mice. CLL or PBS were administered 24 hours prior to infection with 10⁸ FITC-labeled leptospires, followed by liver imaging at 10 min p.i. **(L)** Representative still frames from IVM videos showing the liver sinusoids of control (left) and CLL-treated (right) mice. Leptospires (green), KCs (anti-F4/80, red), and endothelial cells (blue) are shown. KC-associated leptospires (Lep) per ﬁeld of view (FOV) were presented as mean ± SEM 3 images. Scale bar, 10 µm. Data are presented as mean ± SEM. Statistical significance was determined with Student’s t test (unpaired, two-tailed). **p < 0.01, ***p < 0.001.

To directly assess the functional contribution of liver macrophages to the early clearance of leptospires, we depleted this cell population using clodronate liposomes (CLL) (Fig 3A). Compared to control mice, CLL-treated mice exhibited a significantly reduced liver leptospiral load at 10, 30, and 60 minutes post-infection (Fig 3B–3D). By contrast, the blood leptospiral load in mice of the CLL-treated group was significantly higher than that in the control group at 30 min and 60 min after infection with *Leptospira* (Fig 3E–3G). Consistent with the above observations, IF staining of liver sections further confirmed that leptospiral loads were significantly diminished in CLL-treated mice relative to controls at every tested time point (Fig 3H–3J). To visualize this defect in real-time, we performed IVM (Fig 3K). In control mice, leptospires were rapidly immobilized by KCs within the liver sinusoids within 10 minutes of infection. In stark contrast, significantly fewer leptospires were captured in the livers of macrophage-depleted mice, with most leptospires remaining motile in the circulation (Fig 3L and S2 Movie). Collectively, these data demonstrate that liver macrophages are essential for the rapid capture of leptospires from the bloodstream during the very early intravascular phase of infection.

### *L. interrogans* infection enhances the expression of the pattern recognition receptor *Clec4d* in liver macrophages

**Fig 4:**
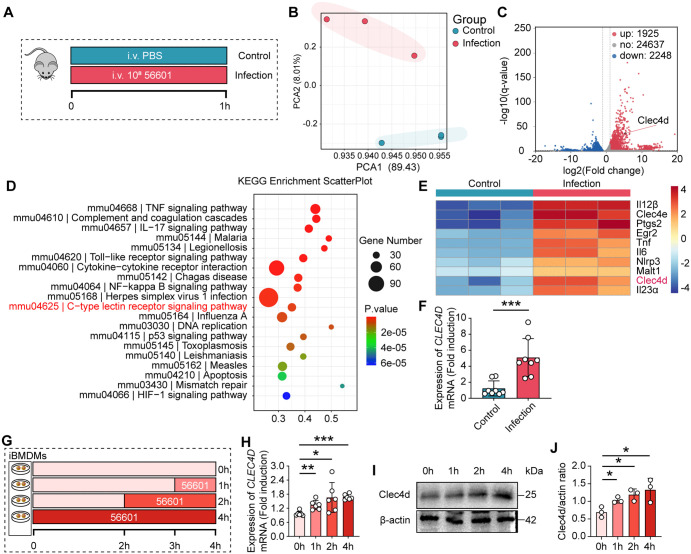
Transcriptomic profiling identifies *Clec4d* as a key upregulated receptor in KCs following *L. interrogans* infection. **(A)** Schematic of the RNA sequencing (RNA-seq) experimental workflow. KCs were isolated from the livers of male C57BL/6 mice at 60 min post-infection with 10⁸ leptospires or from uninfected controls for transcriptome analysis. **(B)** Principal component analysis (PCA) of the transcriptomes from uninfected and infected KCs. **(C)** Volcano plot of differentially expressed genes. Each dot represents a gene. Red dots indicate significantly upregulated genes (log₂FC > 1, p < 0.05), blue dots indicate downregulated genes, and gray dots indicate non-significant changes. Clec4d is highlighted and labeled. **(D)** Enrichment analysis of the most significantly altered signaling pathways from the KEGG database. **(E)** Heatmap showing the expression patterns of enriched differential genes in the C-type lectin receptor signaling pathway. **(F)** Validation of Clec4d upregulation by qPCR in KCs after infection (n = 8). (**G**) iBMDMs were infected with *L. interrogans* for 1, 2, and 4 hours. Total RNA and protein were harvested and analyzed for Clec4d expression. **(H)** Clec4d mRNA expression in iBMDMs at 1, 2, and 4hours post-infection (p.i.) by qPCR (n = 6). **(I)** Representative western blot showing Clec4d protein levels in iBMDMs at the indicated times p.i. **(J)** Densitometric quantification of Clec4d protein levels from **(I)**, normalized to a loading control (n = 3). Data are presented as mean ± SEM. Statistical significance was determined with Student’s t test (unpaired, two-tailed). *p < 0.05, **p < 0.01, ***p < 0.001.

Macrophages are activated and phagocytose pathogens upon recognition of conserved microbial structures by their surface pattern recognition receptors (PRRs). To further investigate the mechanisms by which KCs recognize and capture leptospires, we isolated KCs from the livers of mice at 60 minutes post-infection and performed RNA sequencing (RNA-seq) analysis (Fig 4A). A significant difference in the transcriptome was observed between the control group and the infection group (Fig 4B). A volcano plot depicted the transcriptional changes in all genes. Compared with the control group, the infection group showed 2248 significantly downregulated genes and 1925 significantly upregulated genes (Fig 4C). KEGG pathway analysis revealed that the C-type lectin receptor signaling pathway was significantly enriched in the infection group (Fig 4D). Notably, among the 10 significantly altered genes in the C-type lectin receptor signaling pathway, *Clec4d* exhibited pronounced upregulation in the infection group (Fig 4E). To confirm this transcriptomic observation, we isolated KCs from mice infected with *Leptospira* at 1 hour post-infection, alongside KCs from uninfected control mice, and validated *Clec4d* expression via qPCR. The results showed that compared with the control group, KCs from infected mice exhibited a significant increase in *Clec4d* gene expression (Fig 4F). To investigate Clec4d expression in a different macrophage model, we infected iBMDMs with *L. interrogans* (Fig 4G). *Clec4d* expression was significantly upregulated at 1, 2, and 4 hours post-infection, as detected by qPCR (Fig 4H). Besides, Clec4d protein levels were correspondingly increased, as demonstrated by Western blot analysis (Fig 4I and 4J). In addition to the C-type lectin receptor signaling pathway, KEGG analysis also revealed significant enrichment of the cytokine–cytokine receptor interaction pathway, with the TNF signaling pathway being the most prominently affected. To directly assess functional involvement, we pretreated mice with a neutralizing anti-TNF-α antibody (MP6-XT22) prior to infection and quantified hepatic leptospiral loads at 30 min post-infection. No significant difference was observed between the anti-TNF-α-treated group and the control group (S5E–S5G Fig), indicating that TNF-α does not mediate early leptospiral capture during the first hour of infection.

Collectively, these results indicate that while the C-type lectin receptor signaling pathway, particularly Clec4d, is rapidly upregulated and may play a direct role in leptospiral recognition, the enrichment of cytokine-related pathways primarily reflects secondary inflammatory activation rather than a direct mechanism driving bacterial clearance during the very early intravascular phase.

### Clec4d is required for liver macrophages to capture leptospires

**Fig 5:**
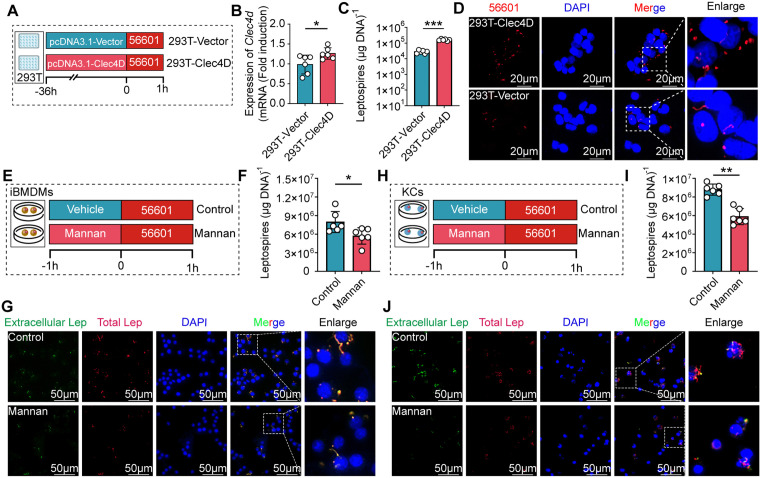
Clec4d is necessary and sufficient for the capture of leptospires. **(A)** Schematic of the ectopic expression assay. HEK-293T cells were transfected to overexpress Clec4d (293T-Clec4D) or with an empty vector control (293T-Vector). Cells were infected with leptospires for 1 hour, followed by washing to remove non-internalized leptospires, and then processed for qPCR and IF analysis of the capture of leptospires. **(B)** Quantitative real-time polymerase chain reaction (qPCR) was used to quantify the expression of *Clec4d* in 293T-Vector and 293T-Clec4D (n = 6). **(C)** Quantification of internalized leptospires in control and Clec4d-overexpressing HEK-293T cells by qPCR (n = 6). **(D)** Representative IF images showing captured leptospires in control and Clec4d-overexpressing HEK-293T cells. Scale bar, 20 µm. **(E)** Schematic of the Clec4d inhibition assay in iBMDMs. Cells were pretreated with mannan or vehicle control for 1 hour; then infected with leptospires for 1 hour. Leptospires undergoing phagocytosis were quantified by qPCR and IF. **(F)** Quantification of internalized leptospires in control and mannan-treated iBMDMs by qPCR (n = 6). **(G)** Representative IF images showing captured *Leptospira* in control and mannan-treated iBMDMs. Scale bar, 50 µm. **(H)** Schematic of the Clec4d inhibition assay in KCs. Primary KCs were pre-treated with mannan or a control for 1 hour prior to a 1-hour infection. Internalized leptospires were quantified. **(I)** Quantification of internalized leptospires in control and mannan-treated KCs by qPCR (n = 6). **(J)** Representative IF images showing captured leptospires in control and mannan-treated KCs. Scale bar, 50 µm. Data are presented as mean ± SEM. Statistical significance was determined with Student’s t test (unpaired, two-tailed). *p < 0.05, **p < 0.01, ***p < 0.001.

**Fig 6:**
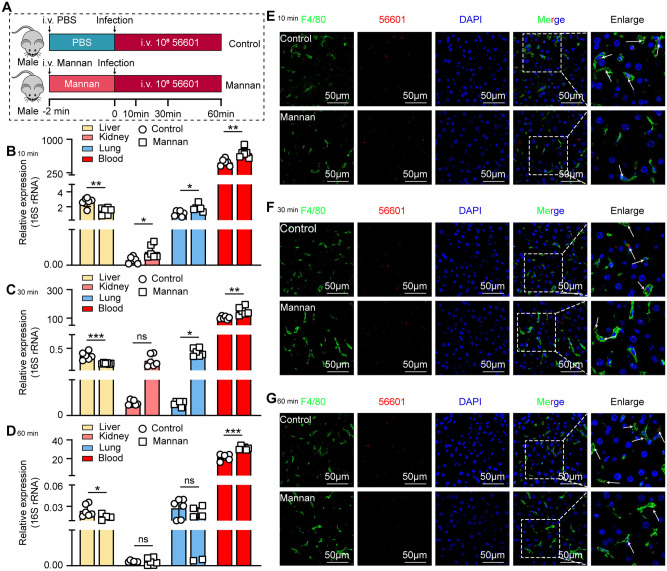
Inhibition of Clec4d impairs liver capture and promotes systemic dissemination of leptospires. **(A)** Schematic of the *in vivo* Clec4d blockade experiment. Mice were intravenously administered the Clec4d antagonist mannan or a control 2 min prior to infection with *Leptospira*. Livers were collected at 10, 30, and 60 min post-infection (p.i.) for analysis. **(B-D)** Leptospiral load in the liver, kidneys, lungs, and blood of control and mannan-treated mice at 10 min **(B)**, 30 min **(C)**, and 60 min (D) p.i., as determined by qPCR (n = 6). **(E-G)** Representative IF images of liver sections from control and mannan-treated mice at 10 min **(E)**, 30 min **(F)**, and 60 min (G) p.i., showing the co-localization of macrophages (anti-F4/80, green) with leptospires (red). Nuclei are stained with DAPI (blue). Scale bars, 50 µm. Arrows indicate leptospires colocalized with macrophages. Data are presented as mean ± SEM. Statistical significance was determined with Student’s t test (unpaired, two-tailed). *p < 0.05, **p < 0.01, ***p < 0.001, ns = non-significant.

Based on the RNA-seq findings suggesting a potential role for Clec4d in the recognition of leptospires by KCs, we next sought to determine whether Clec4d directly mediates the capture of leptospires by these liver-resident macrophages. We ectopically expressed Clec4d in HEK-293T cells to evaluate its direct role in mediating leptospires capture (Fig 5A). As expected, Clec4d-overexpressing HEK-293T cells captured a significantly higher number of leptospires compared to control cells (Fig 5B–5C). Similarly, IF analysis confirmed that Clec4d overexpression led to a marked increase in the capture of leptospires by HEK-293T cells (Fig 5D). Next, we utilized mannan, a competitive antagonist of Clec4d, to assess the impact of receptor blockade on the phagocytic capability of macrophages against leptospires (Fig 5E and 5H). Accordingly, results showed that pretreatment with mannan significantly reduced the number of leptospires captured by both iBMDMs and KCs *in vitro* (Fig 5F and 5I). Similarly, IF images analysis confirmed that mannan treatment led to a marked reduction in the capture of leptospires by both iBMDMs and KCs (Fig 5G and 5J). Then, we explored the effect of mannan on the liver’s ability to capture leptospires *in vivo* (Fig 6A). The liver leptospiral load in mannan-administered mice was significantly reduced relative to control mice at 10 min (Fig 6B), 30 min (Fig 6C), and 60 min (Fig 6D). IF analysis revealed a marked reduction in leptospiral capture by liver macrophages in the mannan-treated group (Fig 6E–6G). Complement C3 and C5 are central to host defense against *Leptospira* [23,24]. Studies have shown that deficiency of either component severely impairs bacterial clearance. C3‑knockout mice exhibit significantly higher renal leptospiral loads and increased renal fibrosis after infection [24]. Similarly, C5‑deficient mice display elevated hepatic leptospiral burdens during early infection [23]. To determine whether mannan treatment activates the lectin pathway of complement and thereby influences leptospiral capture, we measured serum complement C3 and C5 levels in mannan‑treated mice. In the absence of infection, serum C3 levels showed no significant differences between mannan‑treated and control groups at any time point (S6B Fig). For C5, no significant difference was detected at 2 min, 1 h, or 2 h; however, at 4 h post‑injection, C5 levels were significantly elevated in mannan‑treated mice compared to controls (S6C Fig). During *Leptospira* infection, at 10, 30, and 60 min post‑infection, neither C3 nor C5 levels differed significantly between mannan‑treated and control groups (S6E–S6J Fig). Since all of our functional readouts for leptospiral capture were performed within the first hour post‑infection (10, 30, and 60 min), the absence of complement activation at these time points indicates that the impaired leptospiral capture in mannan‑treated mice cannot be attributed to complement‑mediated effects. Rather, it specifically reflects acute blockade of Clec4d. Therefore, our data establish Clec4d as a critical capture receptor in the liver, whose functional integrity is crucial for preventing the systemic dissemination of leptospires by ensuring their early clearance from the bloodstream.

### *L. interrogans* infection induces *Clec4d* expression in liver macrophages via C/EBPβ

**Fig 7:**
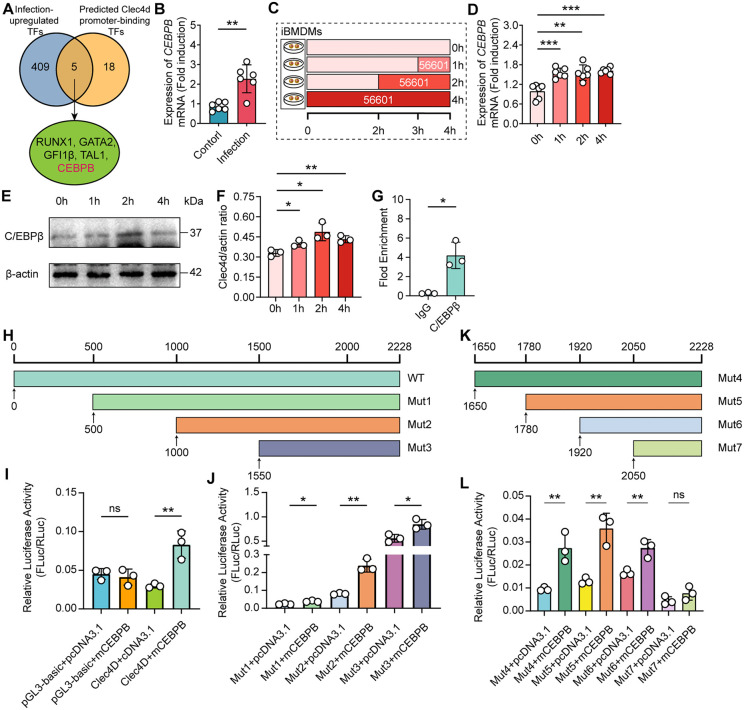
The transcription factor C/EBPβ is activated by infection and directly transactivates the Clec4d gene. **(A)** Venn diagram showing the intersection between transcription factors (TFs) upregulated in KCs after *L. interrogans* infection (from RNA-seq) and TFs predicted to bind the Clec4d promoter (from the ChEA Transcription Factor Targets database). **(B)** Validation of C/EBPβ upregulation by qPCR in KCs after 1 hour of infection (n = 6). **(C)** Schematic of the *in vitro* time-course experiment. iBMDMs were infected with leptospires for 1, 2, and 4 hours, followed by collection of total RNA and protein. **(D)** C/EBPβ mRNA expression in iBMDMs at 1, 2, and 4 hours post-infection (p.i.) by qPCR (n = 6). **(E)** Representative western blot showing C/EBPβ protein levels in iBMDMs at the indicated times p.i. **(F)** Densitometric quantification of C/EBPβ protein levels from **(E)**, normalized to a loading control (n = 3). **(G)** ChIP-qPCR analysis showing the enrichment of the Clec4d promoter region immunoprecipitated with an anti-C/EBPβ antibody relative to control IgG (n = 3). **(H)** Schematic of the dual-luciferase reporter assay using full-length and truncated Clec4d promoter constructs (wild-type WT, Mut1, Mut2, Mut3). **(I)** Relative luciferase activity of the WT Clec4d promoter upon co-transfection with a C/EBPβ expression vector or empty vector control (n = 3). **(J)** Relative luciferase activity of the truncated Clec4d promoter constructs (Mut1-Mut3) upon co-transfection with C/EBPβ (n = 3). **(K)** Schematic of the refined dual-luciferase reporter assay using further truncated Clec4d promoter constructs (Mut4, Mut5, Mut6, Mut7). **(L)** Relative luciferase activity of the further truncated Clec4d promoter constructs (Mut4-Mut7) upon co-transfection with C/EBPβ (n = 3). Data are presented as mean ± SEM. Statistical significance was determined with Student’s t test (unpaired, two-tailed). *p < 0.05, **p < 0.01, ***p < 0.001, ns = non-significant.

Given that gene expression is predominantly regulated by transcription factors (TFs) [25], we next aimed to elucidate the mechanisms underlying *Clec4d* upregulation in response to *L. interrogans* infection by identifying the specific TFs responsible for driving its transcriptional activation. We first employed the ChEA Transcription Factor Targets database to computationally predict TFs potentially regulating Clec4d (S2 Table). This prediction was then integrated with our RNA-seq data, focusing on TFs that were themselves significantly upregulated upon infection (S3 Table). Prompted by its status as the most highly expressed TF (highest FPKM value), we hypothesized that CCAAT/enhancer-binding protein β (C/EBPβ) is a key candidate transcriptional regulator of the Clec4d gene during *Leptospira* infection (Fig 7A). To validate the RNA-seq finding that *Cebpb* is transcriptionally upregulated in liver KCs during *L. interrogans* infection, we first analyzed *Cebpb* mRNA expression in KCs isolated from the livers of infected and control mice. The result showed that *Cebpb* mRNA expression in KCs from the infection group was significantly higher than that in the control group (Fig 7B). To further confirm this infection-induced regulatory pattern, we extended our assessment to iBMDMs (Fig 7C). The result showed that *L. interrogans* infection significantly upregulated C/EBPβ mRNA in iBMDMs (Fig 7D). And, Western blot analysis demonstrated a corresponding increase in C/EBPβ protein levels (Fig 7E and 7F). We next sought to elucidate whether C/EBPβ directly regulates Clec4d transcription by targeting its promoter region. Using iBMDMs as the experimental model, we performed chromatin immunoprecipitation coupled with quantitative polymerase chain reaction (ChIP-qPCR) and verified that C/EBPβ was capable of binding to the Clec4d promoter to modulate its transcriptional activity (Fig 7G). To further determine whether C/EBPβ activates the transcription of *Clec4d*, we performed a luciferase reporter assay (Fig 7H). The results demonstrated that C/EBPβ enhanced the luciferase (LUC) expression driven by the *Clec4d* promoter, suggesting its role in the transcriptional activation of the *Clec4d* gene (Fig 7I). To precisely map the C/EBPβ binding site within the promoter, we generated a series of sequential 5’ deletion constructs: Mut1 (500–2228 bp), Mut2 (1000–2228 bp), and Mut3 (1550–2228 bp). The Mut3 construct retained full responsiveness to C/EBPβ, localizing the critical region to the 1550–2228 bp interval (Fig 7J). We further refined this region using four additional truncations: Mut4 (1650–2228 bp), Mut5 (1780–2228 bp), Mut6 (1920–2228 bp), and Mut7 (2050–2228 bp) (Fig 7K). Luciferase activity was retained in Mut6 but abolished in Mut7 (Fig 7L), demonstrating that the 1920–2050 bp region is essential for C/EBPβ-mediated transactivation of the *Clec4d* promoter.

**Fig 8:**
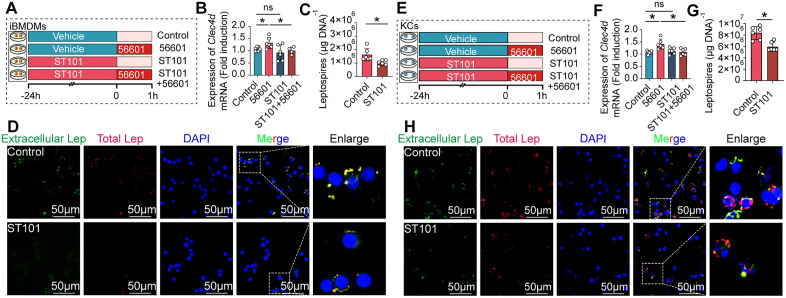
C/EBPβ is required for the infection-induced upregulation of Clec4d and subsequent capture of leptospires in macrophages. **(A)** Schematic of the *in vitro* C/EBPβ inhibition assay in iBMDMs. Cells were pre-treated with the C/EBPβ inhibitor ST101 or vehicle control for 24 hours, followed by infection (or not) with *L. interrogans* strain 56601 for 1 hour where indicated. Cells were then processed for qPCR analysis of Clec4d expression, and for quantification of the capture of leptospires by qPCR and IF. **(B)** *Clec4d* mRNA expression levels in iBMDMs across the four treatment groups: control, 56601 infections alone, ST101 treatment alone, and ST101 pretreatment followed by infection (ST101+ 56601), as determined by qPCR (n = 6). **(C)** Quantification of internalized leptospires in iBMDMs for ST101 treatment groups and control groups, as measured by qPCR (n = 6). **(D)** Representative IF images showing internalized leptospires (red) in iBMDMs for ST101 treatment groups and control groups. Macrophages are stained with an anti-F4/80 antibody (green), and nuclei with DAPI (blue). Scale bar, 50 µm. **(E)** Schematic of the parallel C/EBPβ inhibition assay performed in primary KCs, as described in **(A)**. **(F)** *Clec4d* mRNA expression levels in KCs across the four treatment groups, as determined by qPCR (n = 6). **(G)** Quantification of internalized leptospires in KCs for ST101 treatment groups and control groups, as measured by qPCR (n = 6). **(H)** Representative IF images showing internalized leptospires (red) in KCs for ST101 treatment groups and control groups. Macrophages are stained with an anti-F4/80 antibody (green), and nuclei with DAPI (blue). Scale bar, 50 µm. Data are presented as mean ± SEM. Statistical significance was determined with Student’s t test (unpaired, two-tailed). *p < 0.05, ns = non-significant.

Having identified a functional C/EBPβ binding site in the Clec4d promoter, we next asked whether this regulation is critical during infection. Accordingly, we employed ST101, a specific C/EBPβ antagonist, to assess the impact of C/EBPβ inhibition on *Clec4d* expression and subsequent macrophage phagocytic capability (Fig 8A and 8E). As expected, pretreatment with ST101 significantly reduced *Clec4d* expression in iBMDMs after *L. interrogans* infection (Fig 8B and 8F). Consistent with this finding, ST101 pretreatment markedly reduced the number of leptospires captured by both iBMDMs and KCs *in vitro* (Fig 8C and 8G). This impairment in phagocytic capacity was further confirmed by IF analysis, which showed a substantial decrease in leptospiral internalization by ST101-treated macrophages (Fig 8D and 8H).

**Fig 9:**
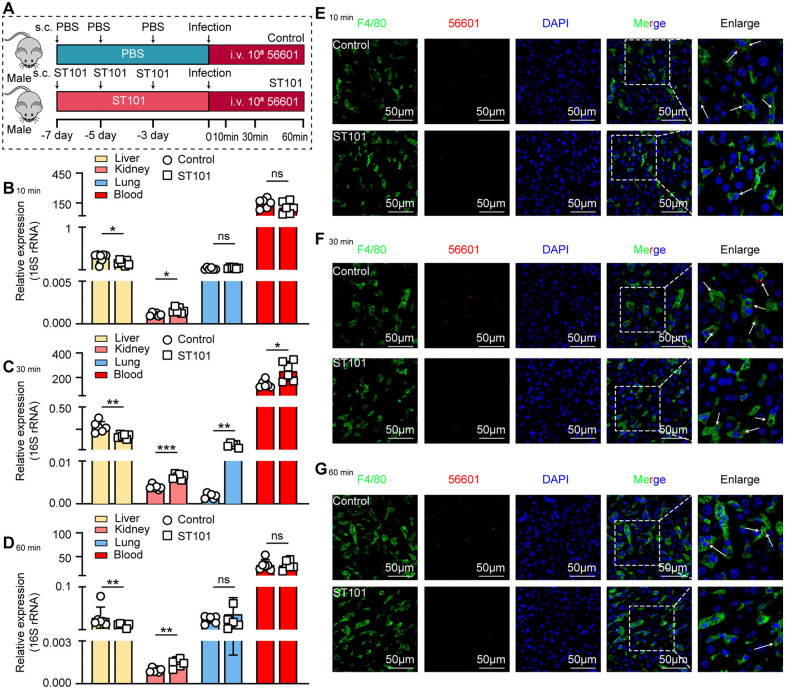
Inhibition of C/EBPβ impairs Clec4d-mediated liver capture and promotes systemic dissemination of leptospires. **(A)** Schematic of the *in vivo* C/EBPβ inhibition experiment. Mice were administered the C/EBPβ inhibitor ST101 (25 mg/kg, s.c.) or vehicle control three times per week for one week prior to intravenous infection with leptospires. Livers were collected at 10, 30, and 60 min post-infection (p.i.) for analysis. **(B-D)** Leptospiral load in the liver, kidneys, lungs and blood of control and ST101-treated mice at 10 min **(B)**, 30 min **(C)**, and 60 min (D) p.i., as determined by qPCR (n = 6). **(E-G)** Representative IF images of liver sections from control and ST101-treated mice at 10 min **(E)**, 30 min **(F)**, and 60 min (G) p.i., showing the co-localization of macrophages (anti-F4/80, green) with leptospires (red). Nuclei are stained with DAPI (blue). Scale bars, 50 µm. Arrows indicate leptospires colocalized with macrophages. Data are presented as mean ± SEM. Statistical significance was determined with Student’s t test (unpaired, two-tailed). *p < 0.05, **p < 0.01, ***p < 0.001, ns = non-significant.

We further examined whether C/EBPβ participated in the capture of *Leptospira* by macrophages *in vivo.* (Fig 9A). The results showed that the leptospiral load in the livers of ST101-treated mice was significantly lower than that in the control group at 10 min (Fig 9B), 30 min (Fig 9C), and 60 min (Fig 9D). IF analysis revealed that ST101 significantly reduced the colocalization of macrophages and leptospires in the liver. (Fig 9E–9G). Collectively, these results demonstrate that C/EBPβ-mediated transcriptional upregulation of *Clec4d* is essential for the capture of leptospires by liver macrophages.

## Discussion

The liver has long been recognized as a central hub for filtering circulating pathogens and orchestrating systemic innate immunity [26], yet its role in the early defense against leptospirosis has remained a critical blind spot in the field. Prior investigations into leptospiral pathogenesis have overwhelmingly centered on renal colonization and late-stage organ damage [27,28]. Studies have demonstrated that the degree of control achieved during the early stages of leptospiral infection determines the extent of subsequent renal colonization by the pathogen [6,29]. However, the mechanisms by which the host captures *Leptospira* during the early stage of infection remain unclear. In this study, we fill this gap by unveiling a previously unappreciated innate immune axis centered on liver KCs, which rapidly sequester leptospires from the circulation via the C/EBPβ-Clec4d transcriptional and receptor cascade. The host innate immune response to *Leptospira* involves coordinated actions of the complement system, pattern recognition receptors (PRRs), and phagocytes. Complement activation facilitates opsonization and enhances bacterial clearance, while pathogenic leptospires evade this defense through multiple mechanisms [30–32]. Beyond complement, Toll-like receptors (TLR2, TLR4) and NOD-like receptors recognize leptospiral components and trigger pro-inflammatory cytokine production [8,9]. These receptors are critical for activating macrophages and neutrophils, the primary effector cells for leptospiral phagocytosis. Our identification of Clec4d as a direct capture receptor adds to this PRR repertoire, highlighting the cooperative roles of different recognition systems in early anti-leptospiral immunity. This discovery not only redefines the spatial and temporal landscape of early leptospiral defense but also establishes a new framework for understanding how tissue-resident macrophages tailor their responses to spirochetal pathogens.

Our initial observation is that the liver accumulates a far higher leptospiral load than the spleen, lungs, or kidneys within the first hour of infection. This finding is consistent with a recent short-term murine model study by Surdel et al., which first established that pathogenic *Leptospira* have increased tropism for the liver and kidney at one-hour post-inoculation [28]. Using a different set of *L. interrogans* strains (serovars *Lai, Canicola*, and *Autumnalis*) and a broader range of early time points (10, 30, and 60 min), we further observed that the liver consistently harbored a significantly higher bacterial load than the kidney. This pattern was independent of bacterial strain, host sex, and inoculation route, and persisted after cardiac perfusion. Notably, in the intraperitoneal infection model, the hepatic leptospiral load became significantly higher than that in other organs only at 60 min post‑infection, which is likely because after IP injection bacteria reach the circulation with a delay, explaining why the hepatic predominance became most apparent at 60 min rather than at earlier time points. The consistency of our liver-centric phenotype across multiple experimental conditions strongly confirms the liver as the primary organ for early leptospiral clearance.

Macrophages are one of the most critical innate immune cells for eliminating invading pathogens [11,26,33]. Studies have shown that liver macrophages, in particular Kupffer cells, can capture circulating bacteria from the bloodstream, acting as a first line of defense in the liver [34,35]. While macrophages are known to contribute to the host response in leptospirosis, their specific role in the initial physical clearance of leptospires from the bloodstream has remained undefined. Our combined flow cytometric, immunofluorescence, and IVM data definitively pinpointed KCs as the dominant mediators of early leptospiral capture. Notably, the negligible contribution of neutrophils to early capture was observed, despite their ability to bind leptospires *in vitro*. The role of neutrophils in leptospirosis is multifaceted and context-dependent. Scharrig et al. demonstrated that neutrophils release extracellular traps (NETs) to entrap leptospires, which contributes to early host defense and prevents bacterial dissemination [36]. In contrast, Shetty et al. reported that neutrophils are not prominently engaged in the early immune response to pathogenic *Leptospira*, suggesting a possible mechanism by which the bacteria evade innate immunity [37]. Moreover, Papadopoulos et al. recently showed that in fatal murine leptospirosis, neutrophilia and neutrophil-mediated vascular leakage, rather than cytokine storm, are associated with disease severity, indicating a potentially deleterious role of neutrophils in leptospirosis pathogenesis [38]. Collectively, these findings suggest that neutrophils may play dual roles in leptospirosis, and their functional contribution likely varies depending on the infection stage, bacterial strain, and host genetic background. Further studies are needed to dissect the precise role of neutrophils in the early stage of leptospiral infection.

The specific mechanisms by which macrophages recognize and phagocytose leptospires have remained poorly defined, despite their crucial role in the early host defense. Clec4d is a C-type lectin receptor predominantly expressed on myeloid cells and functions as a pattern recognition receptor (PRR) of the innate immune system [39]. Previous studies have established a critical role for Clec4d in anti‑mycobacterial immunity, where it recognizes mycobacterial trehalose 6,6‘-dimycolate (TDM, also known as cord factor), a cell wall glycolipid component [29,40,41]. In addition, Clec4d plays a key role in anti‑fungal defense. It recognizes α-mannans on the hyphae of *Candida* albicans and forms heterodimers with Dectin-2, which enhances ligand binding and amplifies inflammatory responses [42,43]. Furthermore, Clec4d has been shown to directly bind glucuronoxylomannan (GXM) from *Cryptococcus neoformans* and *Cryptococcus gattii*, and Clec4d‑deficient mice exhibit increased susceptibility to cryptococcal infection due to impaired alveolar macrophage accumulation and killing [44]. Thus, Clec4d serves as a versatile PRR capable of recognizing diverse carbohydrate structures from both bacteria and fungi. In the present study, our RNA‑seq and functional analyses identified Clec4d as a key capture receptor on Kupffer cells that captures leptospires. Importantly, the Clec4d‑dependent mechanism we identified is likely also operational in other macrophage populations beyond KCs, as mannan treatment impaired leptospiral capture in both primary KCs and iBMDMs (Fig 5F–5J). However, while the ligands for Clec4d in mycobacterial and fungal infections are relatively well defined, the specific leptospiral component(s) recognized by Clec4d remain unknown. Leptospires are spirochetes whose outer membrane is composed of lipopolysaccharide (LPS) and various outer membrane proteins. The glycan structure of leptospiral LPS is highly heterogeneous among different serovars, and its monosaccharide composition differs from that of many other pathogens [45]. Whether leptospires express α‑mannan‑like structures or possess unique glycosylation patterns that are specifically recognized by Clec4d is currently unclear. Thus, deciphering the precise molecular ligand on *Leptospira* that is engaged by Clec4d represents an important direction for future research.

Our RNA‑seq analysis of Kupffer cells revealed significant enrichment of the cytokine–cytokine receptor interaction pathway, with the TNF signaling pathway being the most prominently affected. To determine whether these cytokines actively participate in the early capture of leptospires or instead reflect downstream inflammatory responses, we focused on TNF‑α. Neutralization of TNF‑α prior to infection did not alter hepatic leptospiral loads (S5 Fig). These findings indicate that TNF‑α does not contribute to immediate Kupffer cell‑mediated leptospiral capture. Moreover, our flow cytometry data show that within the first hour, neutrophils account for only a minor fraction of leptospire‑positive cells (Fig 2B–2D), consistent with published reports that neutrophil infiltration becomes prominent only after several hours and is associated with tissue injury rather than early clearance [38]. Thus, the enrichment of cytokine‑related pathways in Kupffer cells at 60 min likely represents a preparatory inflammatory state rather than an immediate effector mechanism. Our data suggest that while TNF‑α and other cytokines may contribute to later‑stage host defense or immunopathology, they do not play a direct role in the rapid clearance of leptospires from the bloodstream. This distinction is important for understanding the hierarchy of innate immune mechanisms that operate during the critical early window following pathogen entry. Future time‑course studies examining chemokine/cytokine production (e.g., CXCL1, CXCL2, IL‑6) and neutrophil recruitment at later time points (e.g., 2–24 h) will be valuable to fully understand the temporal relationship between Clec4d‑mediated capture and downstream inflammation.

Consistent with the impaired hepatic capture, mannan-treated mice exhibited increased leptospiral loads in the blood, lungs, and kidneys at certain time points (Fig 6B–6D). This pattern supports our mechanistic model: blocking Clec4d on Kupffer cells reduces the liver’s ability to sequester leptospires from the circulation, allowing more bacteria to remain in the bloodstream and subsequently disseminate to peripheral organs such as the lungs and kidneys. Thus, the opposing tissue effects of mannan further reinforce the critical role of Clec4d-mediated Kupffer cell capture in limiting systemic leptospiral dissemination. While mannan is widely used as a competitive inhibitor of Clec4d, we acknowledge its limited specificity, as it may also interfere with other C-type lectin receptors. Future studies using Kupffer cell-specific Clec4d knockout mice will be essential to further define its role. C3 and C5 are non‑redundant effector molecules in complement‑mediated defense against *Leptospira*. C3‑knockout mice exhibit higher renal leptospiral loads and increased renal fibrosis [24], whereas C5‑deficient mice display elevated hepatic bacterial burdens during the early stage of infection [23]. Therefore, to exclude the possibility that mannan activates the complement system via the lectin pathway [46], we directly measured serum complement C3 and C5 levels following mannan administration. No significant differences were detected between mannan‑treated and control mice (S6 Fig), indicating that mannan did not activate complement under our experimental conditions. Therefore, the impaired leptospiral capture in mannan‑treated mice can be specifically attributed to Clec4d blockade rather than to off‑target complement activation. In conclusion, the consistent results obtained from complementary approaches—including receptor overexpression in non-phagocytic cells, pharmacological blockade, and in vivo inhibition—collectively support a critical role for Clec4d in mediating the recognition and capture of *L. interrogans* by macrophages.

Having established Clec4d as a critical receptor for macrophage-mediated capture of leptospires, we next sought to determine how its expression was regulated upon infection. To elucidate the upstream signaling events that potentiate Clec4d transcription, we re-analyzed our RNA-seq data integrated with predictions from the ChEA Transcription Factor Targets database. This combined approach identified the transcription factor C/EBPβ as a key candidate regulator. Using a dual-luciferase reporter assay, we confirmed that C/EBPβ could indeed activate the Clec4d promoter. Subsequently, a series of promoter truncation experiments localized the specific C/EBPβ binding site to a region between 1920 and 2050 bp. To define the functional relevance of this axis, we employed the specific C/EBPβ inhibitor ST101. Inhibition of C/EBPβ not only abrogated the infection-induced upregulation of Clec4d in macrophages but also crippled their phagocytic capability *in vitro*. Mirroring the phenotype observed with direct Clec4d blockade, *in vivo* administration of ST101 reduced the liver leptospiral load and diminished co-localization of leptospires with liver macrophages. Collectively, these results demonstrate that the C/EBPβ-Clec4d axis constitutes a coherent inducible program essential for the early innate immune defense, functioning as a transcriptional firewall that orchestrates the rapid capture of leptospires.

Although our analysis focused on the first hour post-infection to capture the earliest events in leptospiral sequestration, the spatiotemporal dynamics of leptospiral distribution inevitably evolve as infection progresses. The kidney ultimately becomes the primary reservoir organ in leptospirosis [47]. Studies in murine models have demonstrated that leptospires can be detected in renal tubules as early as day 2 post-infection, with bacterial loads progressively increasing and peaking between days 5 and 7, coinciding with the onset of mild tubulointerstitial inflammation [3,6]. Our finding that the liver serves as the dominant site for early leptospiral capture establishes a critical initial bottleneck: a substantial proportion of circulating bacteria are rapidly sequestered by hepatic macrophages. However, a subset of leptospires may evade this early hepatic capture, or be captured by macrophages but resist intracellular killing, thereby surviving to subsequently disseminate and establish renal colonization [8]. Importantly, the efficiency of this early hepatic capture may directly influence the magnitude of subsequent renal colonization. A recent study by Ratet et al. demonstrated that the efficiency of early innate immune clearance correlates inversely with the bacterial burden that eventually seeds the kidney [6]. Thus, our observation that the liver serves as the predominant organ for early leptospiral capture not only defines an immediate host defense mechanism but also suggests that interventions enhancing this early hepatic sequestration could potentially limit the development of renal carriage—a critical determinant of disease transmission.

While our findings are derived from mouse models, they offer potential insights into human leptospirosis. The liver is a major site of pathology in severe human leptospirosis, manifesting as jaundice and hepatic dysfunction [48]. A critical difference between mice and humans lies in the fate of internalized leptospires. Studies have shown that murine macrophages effectively kill *Leptospira* through reactive oxygen species‑dependent mechanisms, whereas human macrophages permit bacterial survival and even replication [49]. This difference likely explains why humans, as accidental hosts, are more susceptible to severe disease, while mice serve as natural reservoirs that tolerate chronic renal colonization without overt illness.

Importantly, only about 10% of infected individuals develop severe leptospirosis (Weil’s disease), characterized by jaundice, renal failure, and pulmonary hemorrhage [50]. We speculate that in the majority of exposed individuals, Kupffer cell (KC) function remains competent, clearing the infection subclinically. However, in the 10% who progress to severe disease, pre‑existing or concurrent conditions—such as alcohol‑related liver disease, non‑alcoholic steatohepatitis, viral hepatitis, or immunosuppression—may compromise KC phagocytic and microbicidal capacity. For example, chronic liver disease is known to impair KC function, reducing their ability to capture and kill pathogens. When such vulnerable individuals encounter *Leptospira*, the bacteria may evade capture or survive within KCs through mechanisms such as delayed phagosome maturation or induction of KC apoptosis [49], leading to prolonged bacteremia and dissemination. The resulting hepatic injury could involve both direct bacterial effects and KC‑mediated inflammation, ultimately contributing to cholestasis and jaundice.

With respect to jaundice in severe leptospirosis, several mechanisms may link KC dysfunction to hyperbilirubinemia. Activated KCs produce pro‑inflammatory cytokines and reactive oxygen species that can induce hepatocellular injury and intrahepatic cholestasis. Moreover, massive apoptosis of KCs may impair hepatic clearance function and contribute to hepatocyte dysfunction. These mechanisms align with the clinical observation that jaundice in Weil’s disease often correlates more closely with cholestasis than with extensive hepatocellular necrosis [51].

Beyond these cellular mechanisms, inter‑individual variability in the C/EBPβ‑Clec4d axis could plausibly contribute to differences in disease severity. Genetic variations in C‑type lectin receptors have been associated with susceptibility to infectious diseases; for example, a human CLEC4D polymorphism is linked to increased susceptibility to pulmonary tuberculosis [52]. Similarly, polymorphisms in the IL1B promoter that affect C/EBPβ binding influence IL‑1β production and are associated with active tuberculosis [53]. In leptospirosis specifically, polymorphisms in the MIF gene promoter have been shown to determine host susceptibility and disease severity [54], and variants in the complement factor H gene CFH are associated with leptospirosis risk and renal complications [55]. Although direct evidence for CLEC4D or CEBPB polymorphisms in leptospirosis patients is currently lacking, these findings from related infectious diseases suggest that genetic variation in this pathway warrants investigation as a potential determinant of inter‑individual differences in clinical outcomes.

Beyond its theoretical implications, our work provides specific testable hypotheses for host‑directed intervention. For example, pharmacological activation of C/EBPβ (e.g., using small‑molecule agonists) could enhance Clec4d expression and thus improve leptospiral capture by Kupffer cells. Direct agonism of Clec4d (e.g., with specific antibodies or carbohydrate‑based ligands) might similarly boost bacterial internalization. Prophylactic upregulation of Clec4d on liver macrophages prior to exposure could be explored as a preventive strategy in high‑risk populations. We explicitly acknowledge that these concepts remain speculative at this stage, and extensive preclinical development—including the identification of suitable agonists, evaluation of safety and efficacy in animal models, and assessment of potential off‑target effects—would be required before any translational application could be considered.

In conclusion, our study provides a significant advance in understanding the very early innate immune response during the intravascular phase (within the first hour post-infection) to leptospirosis by delineating a previously unappreciated pathway in which the liver, through its resident macrophages, acts as a decisive barrier against systemic dissemination. We demonstrate that this liver defense is mediated by a C/EBPβ-driven, Clec4d-dependent mechanism for the rapid capture of leptospires. These findings fundamentally refine the pathophysiological model of early infection, shifting the paradigm from a predominantly kidney-centric view to one that recognizes the liver’s essential gatekeeper function. Together, our work establishes a new framework for understanding how tissue‑resident macrophages tailor their responses to spirochetal pathogens and provides a foundation for future investigations into host‑directed interventions and genetic susceptibility in human leptospirosis.

## Materials and methods

### Ethics statement

Male and female C57BL/6 mice (8 weeks) were obtained from Liaoning Changsheng Biotechnology Co., Ltd., located in Benxi, China. All mice were maintained at room temperature (20–24°C) and housed on a 12 h light/dark cycle. The mice were used for experiments after a week of adaptive feeding with ample water and breeding fodder. All animal studies received approval from the Institutional Animal Care and Use Committee (IACUC) of Jilin University (SY202508041).

### Mouse infections

Leptospires were counted in the Petroff-Hauser chamber before infection, centrifuged for 10 minutes at 3500 RCF, and resuspended with phosphate buffered saline (PBS) [56]. C57BL/6J mice were injected with 10⁸ leptospires either intraperitoneally or via the tail vein. At the indicated time points post-infection, mice were euthanized for tissue collection. The liver, kidneys, spleen, lungs, and blood were aseptically harvested. For mice subjected to cardiac perfusion, deep anesthesia was induced prior to the procedure. Perfusion was performed by inserting a blunt-ended needle into the left ventricle at the cardiac apex, followed by creating a small incision in the right atrium to serve as an outflow tract. Sterile PBS was perfused at a rate of 5–10 ml/min for 5 minutes. Following perfusion, the liver, kidneys, spleen, and lungs were aseptically collected. All tissue samples were rinsed with PBS to remove residual blood and subsequently stored at -80°C.

### Bacterial strains

The following pathogenic *L. interrogans* strains were used: serogroup Icterohaemorrhagiae serovar Lai strain Lai (56601), serogroup Canicola serovar Canicola strain Lin (56606), and serogroup Autumnalis serovar Autumnalis strain Akiyami A (56603). All strains were grown in liquid Ellinghausen-McCullough-Johnson-Harris (EMJH) medium at 29°C under static conditions. Bacterial concentration was determined by direct counting using a Petroff-Hauser chamber.

### Plasmid construction

The murine Clec4d overexpression plasmid was constructed by cloning the m-Clec4d coding sequence into the pcDNA3.1 vector. The gene fragment was amplified by PCR and inserted into the linearized vector using a restriction-ligation method with BamHI and EcoRI sites. The constructed plasmid was transformed into DH5α competent cells, and positive clones were verified by colony PCR and DNA sequencing. The final plasmid was purified using a commercial kit for subsequent experiments.

The Clec4d promoter and C/EBPβ coding sequence were cloned into pGL3-Basic and pcDNA3.1 vectors, respectively, using standard molecular cloning. Target fragments were amplified by PCR using Pfu DNA polymerase (NEB) with 25 cycles of 95 °C for 15 s, 55 °C for 30 s, and 72 °C for 1 min, followed by gel purification (DNA Gel Extraction Kit, Tiangen). Following double digestion with restriction enzymes (37 °C for 1–2 h), fragments were ligated into linearized vectors using T4 DNA Ligase (Fermentas) (16 °C for 0.5–1 h, with a vector:insert molar ratio of approximately 1:3). The constructs were transformed into TOP10 competent cells (heat‑shock at 42 °C for 90 s, followed by SOC recovery for 45 min at 37 °C), and positive clones were selected by colony PCR and verified by sequencing.

### Cell culture and infection

KCs were isolated from C57BL/6J mice via a modified two-step collagenase perfusion technique [57]. Briefly, mice were anesthetized by intraperitoneal injection of Avertin (600 μL per 25 g body weight). To prevent coagulation, 200 μL of heparin solution (100 U in PBS) was injected intravenously. The liver was perfused in situ through the inferior vena cava using a peristaltic pump at a flow rate of 5 mL/min. First, PBS was perfused for 3 min to remove blood from the liver vasculature. Then, Liver Digestion Medium (Thermo Fisher Scientific) was perfused for 12 min at 37 °C; after approximately 8 min, the liver consistency was checked with a wet cotton applicator until it appeared reticulated. After digestion, the gallbladder was removed, and the liver was transferred to a Petri dish containing Hepatocyte Wash Medium. The organ was gently shaken to release digested cells (hepatocytes and non‑parenchymal cells, LNPCs). The cell suspension was collected and centrifuged at 20 rcf for 3 min to pellet hepatocytes. The supernatant containing LNPCs was transferred to a new tube and centrifuged at 400 rcf for 5 min. The LNPC pellet was resuspended in 36% Percoll solution and centrifuged at 2000 rpm for 20 min without brake to remove debris. After Percoll separation, the LNPC fraction was collected and red blood cells were lysed with ACK buffer for 30 s at room temperature, followed by neutralization with RPMI and centrifugation at 400 rcf for 5 min. KCs were subsequently purified from the non-parenchymal cell fraction using the MojoSort Mouse F4/80 Selection Kit for positive selection of F4/80+ cells. KCs were isolated from liver and cultured in RPMI 1640 supplemented with 10% FCS, and 1% penicillin and streptomycin for 24 h.

iBMDMs were cultured at 37°C and 5% CO2 in Dulbecco’s Modified Eagle’s High Glucose media supplemented with 10% Fetal Bovine Serum.

For the infection assays, cells were seeded at a density of 4 × 10^5^ cells per well. After adherence, the cells were infected with leptospires at a multiplicity of infection (MOI) of 100. Infection times are indicated in the figure legends. Following infection, the cell monolayers were gently washed three times with sterile PBS to remove non-internalized leptospires. Finally, the cells were processed for downstream applications, including the collection of total DNA, RNA, or round glass coverslips for further analysis.

HEK 293T cells were cultured at 37°C and 5% CO2 in Dulbecco’s Modified Eagle’s High Glucose media supplemented with 10% Fetal Bovine Serum. Cells were seeded in 24-well plates at a density of 1 × 10⁵ cells per well 18 hours prior to transfection to achieve 70–80% confluence at the time of transfection. For each well, 2.5 μg of plasmid DNA was diluted in the culture medium and gently mixed. Then, 4 μL of Lipo8000 transfection reagent was added, and the mixture was blended gently by pipetting; vortexing or centrifugation was strictly avoided [58]. The transfection complex was formed at room temperature and added to the cells within 6 hours. After 36 hours of incubation, the cells were either assessed for their ability to capture *Leptospira* or analyzed for relative luciferase activity.

The phagocytosis assay was performed by incubating cells with *L. interrogans* for 1 hour. After incubation, external leptospires were removed by trypsinization followed by three washes in RPMI 1640. Cells were then collected by centrifugation (1200 rpm, 5 min), and the pellets were stored at -80°C for DNA extraction.

### Flow cytometry

To identify immune cells that captured leptospires, captured cells were analyzed using a FACSAria III flow cytometer (BD Biosciences). Single‑cell suspensions of liver non‑parenchymal cells were prepared as described above, and cell concentration was adjusted to 1 × 10⁷ cells/mL in FACS buffer (PBS containing 0.5% BSA and 2 mM EDTA). Cells were incubated with FcR blocking reagent (Miltenyi Biotec) for 15 min at 4 °C to reduce non‑specific binding, followed by staining with fluorochrome‑conjugated antibodies for 30 min at 4 °C in the dark. After staining, cells were washed twice with FACS buffer and resuspended in 300 μL of the same buffer for acquisition. A viability dye (PI) was added to exclude dead cells from analysis. Within the single-cell population, FITC-positive events (indicative of captured leptospires) were first selected. Among these FITC+ cells, leukocytes were gated as CD45+ . Subsequently, specific cell populations were defined as follows: Macrophages that had captured leptospires (pre-labeled with FITC) were identified as having a flow cytometry phenotype of FITC+CD45+ F4/80+ CD11b+ by flow cytometry. At least 100,000 events per sample were acquired, and fluorescence compensation was performed using single‑stained controls and CompBeads (BD Biosciences). Gating strategies were validated using fluorescence‑minus‑one (FMO) controls. In contrast, neutrophils that captured leptospires exhibited a phenotype of FITC+CD45+ Ly6G+F4/80-CD11b+ . Data were analyzed with FlowJo software (Tree Star Inc.).

### *In vivo* treatment of animals with inhibitors

Macrophage ablation was achieved with clodronate liposomes via intravenous injection(i.v.). For pharmacological inhibition of Clec4d, 400 μg of mannan was given i.v. 2 min prior to infection [59]. In the study of C/EBPβ regulating Clec4d expression, lucicebtide (C/EBPβ inhibitor ST101) was administered subcutaneously at 25 mg/kg three times a week for one week [60]. For neutralization of TNF‑α, mice were pretreated with a monoclonal anti‑TNF‑α antibody (MP6‑XT22) via intraperitoneal injection (i.p.) at a dose of 200 μg per mouse 24 h prior to infection, with rat IgG1 isotype control used as a negative control.

### Immunofluorescence (IF) analysis

Liver samples were embedded in optimal cutting temperature compound for IF staining (Servicebio, China). Serial sections of 5 μm thickness were sliced and permeabilized with 0.1% Triton X-100. After being blocked with serum-based blocking buffer for 1 h, the sections were incubated with primary antibodies against F4/80 and leptospires at 4°C overnight. The sections were incubated with FITC-conjugated Goat Anti-Rabbit IgG (H + L) and Cy3-conjugated AffiniPure Goat Anti-Rat IgG (H + L) at 37°C for 1 hour. Counterstaining was performed with 4′,6-Diamidino-2-Phenylindole (DAPI), and a fluorescence microscope was employed for observation and analysis. In IF, cells with red and green overlapping colors are considered as macrophages that had captured *Leptospira*.

For the IF staining of cell climbing slice samples, the following procedure was performed: After infection with *Leptospira*, samples were fixed with 4% paraformaldehyde for 15 min. Following blocking with a serum-based blocking buffer for 1 hour, the samples were incubated with a rabbit-derived anti-*Leptospira* primary antibody at 37°C for 1 hour. Subsequently, an FITC-conjugated goat anti-rabbit IgG (H + L) was applied and incubated at 37°C for 1 hour. Permeabilization was carried out using 0.1% Triton X-100. After another round of blocking with serum-based blocking buffer for 1 hour, the samples were incubated again with a rabbit-derived anti-*Leptospira* primary antibody at 37°C for 1 hour. This was followed by incubation with a Cy3-conjugated goat anti-rat IgG (H + L) at 37°C for 1 hour. Finally, counterstaining was performed with 4′,6-diamidino-2-phenylindole (DAPI), and the samples were observed and analyzed under a fluorescence microscope. In the IF analysis, green signal was interpreted as extracellular leptospires, while red signal was considered to represents both extracellular and intracellular leptospires.

### Western blot

Total protein was obtained using RIPA lysis buffer (Servicebio, China) containing protease/phosphatase inhibitors (NCM Biotech, China). Cells were lysed on ice for 30 min with occasional vortexing, then centrifuged at 12,000 × g for 15 min at 4 °C to collect supernatants. Protein concentration was assessed using a BCA Protein Assay Kit (Thermo Fisher Scientific, USA). Equal amounts of protein (20 μg per sample) were mixed with 5 × SDS loading buffer, boiled at 95 °C for 10 min, and resolved by 10% SDS‑PAGE (Bio‑Rad) at 120 V for 90 min. Proteins were then transferred onto a PVDF membrane (Millipore) using a wet transfer system (Bio‑Rad) at 250 mA for 2 h at 4 °C. After blocking with 5% non‑fat milk in TBST (20 mM Tris‑HCl pH 7.6, 150 mM NaCl, 0.1% Tween‑20) for 1 h at room temperature, the membranes were incubated overnight at 4 °C with primary antibodies diluted in blocking buffer (anti‑Clec4d, 1:1000; anti‑GAPDH, 1:5000). After three washes (10 min each) with TBST, membranes were incubated with HRP‑conjugated secondary antibodies (1:5000 dilution) at room temperature for 1 h, followed by three additional washes. Bands were detected using enhanced chemiluminescent substrates (Thermo Fisher Scientific) and imaged with a ChemiDoc MP system (Bio‑Rad). Densitometric quantification was performed using ImageJ software (NIH), with target protein band intensities normalized to GAPDH as a loading control [61].

### RNA-sequencing analyses

RNA-seq analysis of gene expression: The mRNA sequencing was performed at LC-BIO Co., Ltd (HangZhou, China). Kupffer cells were isolated from mouse livers as described in the Cell culture and infection section. Total RNA was then extracted from these purified Kupffer cells using TRIzol reagent (TaKaRa, Japan). Sequencing libraries were constructed from low-input RNA using the SMART-Seq v4 Ultra Low Input RNA Kit (Clontech, Japan). The process involved cDNA synthesis, followed by purification and size selection (150–300 bp). Sequencing-ready libraries were then prepared via PCR-mediated addition of i5 and i7 adapters, with quality control (size distribution and concentration) assessed on an Agilent 2100 Bioanalyzer, prior to being sequenced on an Illumina platform under a 2x150bp paired-end protocol. For bioinformatics analysis, raw reads were first processed with Cutadapt (version 1.9) to remove adapter contamination. After filtering out low-quality and undetermined bases, the clean reads were aligned to the reference genome (Mus musculus, Ensembl release 96) using HISAT2 (version 2.0.4). Transcript assembly for each sample was performed with StringTie (version 1.3.4d), and expression levels were quantified in FPKM. Differential expression analysis of mRNAs was conducted using edgeR, applying thresholds of fold change > 2 and p-value < 0.05. Finally, the differentially expressed mRNAs were functionally annotated through Gene Ontology (GO) and Kyoto Encyclopedia of Genes and Genomes (KEGG) pathway analyses.

### Dual luciferase activity assays

The LUC and REN activities were determined according to the instructions of the Dual Luciferase Reporter Gene Assay Kit (Mei5bio, China). Briefly, 10–20 μL of cell lysate was transferred into an opaque 96‑well plate. Then, 100 μL of Luciferase Reaction Reagent (equilibrated to room temperature) was added to each well, and the plate was mixed by horizontal shaking. Firefly luciferase activity was measured using a luminometer at an emission wavelength of 350–700 nm with a detection time of 1 second per well. If the reading reached the upper limit, the volume of lysate was reduced appropriately. Subsequently, 100 μL of Luciferase Reaction Reagent II (equilibrated to room temperature) was added to the same wells, mixed by horizontal shaking, and Renilla luciferase activity was measured at 380–780 nm for 1 second per well. The relative promoter activity was expressed as the ratio of firefly luminescence to Renilla luminescence (LUC/REN).

### ChIP-qPCR

The binding of C/EBPβ to the Clec4d promoter was analyzed by ChIP. iBMDMs (1 × 10⁷) were crosslinked with 1% formaldehyde for 10 min, quenched with glycine, and lysed. Nuclei were collected and chromatin was sonicated to 200–500 bp fragments. After dilution, chromatin was immunoprecipitated overnight at 4 °C with 3 µg of anti‑C/EBPβ antibody or normal rabbit IgG (negative control). Protein A/G magnetic beads were added for 2 h, followed by sequential washes (low‑salt, high‑salt, LiCl, TE). Immunocomplexes were eluted and crosslinking was reversed by overnight incubation at 65 °C with 200 mM NaCl. DNA was purified, and qPCR was performed with primers specific for the Clec4d promoter. Enrichment was calculated as percent input using the formula: % Input = 100 × 2 ^ [Ct (Input) – Ct (IP)].

### Extraction of total RNA and Real-Time PCR (qPCR)

Total RNA was extracted from tissues (liver, kidney, spleen, lung) and blood using TRIzol reagent (Invitrogen, USA) according to the manufacturer’s instructions. RNA was reverse-transcribed into cDNA using an ABScript III RT Master Mix with gDNA Remover [62]. Quantitative real-time PCR (qPCR) was performed on a CFX Connect Real-Time System (Bio-Rad, USA) using SYBR Premix EsTaq. To quantify leptospiral load in tissues, qPCR targeting the 16S rRNA gene of *Leptospira interrogans* was used [63,64]. The primer sequences were forward: 5′- AGCACGTGTGTTGCCCTAGACATA-3′ and reverse: 5′- GTTGCCATCATTCAGTTGGGCACT-3′. For each sample, the Ct value was normalized to tissue weight (1 mg) or blood volume (200 μL) to account for differences in sample input. Results were expressed as relative leptospiral load (2 ⁻ Δᶜᵗ) normalized to a reference sample. The mRNA levels of host target genes were quantified and normalized to those of *GAPDH*. Primer sequences used for host gene detection are presented in S1 Table.

### Measurement of serum complement C3 and C5 levels

Mouse serum samples were collected at indicated time points post‑infection and stored at −80 °C until analysis. Serum complement C3 (C3) levels were measured using a Mouse C3 ELISA Kit (Solarbio Beijing, China) following the manufacturer’s instructions. Serum complement C5 (C5) levels were measured using a Mouse Complement Component C5 ELISA Kit (Solarbio, Beijing, China). Standards (prepared by serial dilution according to the kit instructions) and pre‑diluted serum samples (diluted 1:10000 for C3 and 1:100 for C5 in the provided sample diluent) were added to the appropriate wells (100 μL/well) and incubated at 37 °C for 1.5 h (C3) or 1.5 h (C5). After incubation, the wells were aspirated and washed five times with wash buffer (350 μL/well). Subsequently, 100 μL of biotinylated detection antibody working solution was added to each well and incubated at 37 °C for 1 h. Following another wash step, 100 μL of horseradish peroxidase (HRP)‑conjugated streptavidin working solution was added and incubated at 37 °C for 30 min. After a final wash, 100 μL of TMB substrate solution was added to each well and incubated at 37 °C in the dark for 15 min. The reaction was terminated by adding 50 μL of stop solution, and the absorbance was read immediately at 450 nm using a microplate reader. A standard curve was generated by plotting the absorbance values of the standards against their concentrations, and the sample concentrations were calculated from the standard curve using four‑parameter logistic regression.

### Statistics

Statistical differences among the indicated groups were analyzed by unpaired two-tailed Student’s t-test or one-way or two-way analysis of variance (ANOVA) using Tukey’s multiple comparison test. All statistical analyses were done using GraphPad Prism 9. A *p* value of < 0.05 was considered significant. Sample sizes (n) and other relevant values are indicated in the figures and figure legends (*: *p* < 0.05; **: *p* < 0.01; ***: *p* < 0.001; ns = non-significant).

## Supporting information

S1 Fig The liver is the primary organ for capturing leptospires during the early phase of infection in both sexes. **(A)** Experimental schematic for determining bacterial distribution in female mice. Female C57BL/6 mice were intravenously infected with 10⁸ *L. interrogans* serovar Lai (strain 56601). Bacterial loads in the liver, kidneys, spleen, lungs, and blood were quantified by qPCR at 10, 30, and 60 minutes post-infection (p.i.). **(B-D)** In each graph, the bars from left to right represent the leptospiral loads in the blood, liver, kidneys, spleen, and lungs at 10 min **(B)**, 30 min **(C)**, and 60 min **(D)** p.i. (n = 6). Data are presented as mean ± SEM. Statistical significance was determined with Student’s t test (unpaired, two-tailed). *p < 0.05, **p < 0.01, ***p < 0.001.
(TIF)

S2 Fig The liver is the primary organ for capturing leptospires during the early phase of infection after cardiac perfusion. **(A)** Experimental schematic for determining bacterial distribution after cardiac perfusion in male mice. C57BL/6 mice were intravenously infected with 10⁸ *L. interrogans* serovar Lai (strain 56601). Prior to tissue collection at 10, 30, and 60 minutes p.i., mice underwent rigorous cardiac perfusion with sterile PBS to eliminate blood from the tissue vasculature. (**B-D**) In each graph, the bars from left to right represent the leptospiral loads in the liver, kidneys, spleen, and lungs of perfused male mice at 10 min **(B)**, 30 min **(C)**, and 60 min **(D)** p.i. (n = 6). Data are presented as mean ± SEM. Statistical significance was determined with Student’s t test (unpaired, two-tailed). *p < 0.05, ***p < 0.001.
(TIF)

S3 Fig Gating strategy for flow cytometric identification of Kupffer cells and neutrophils in the liver. **(A)** Gating strategy for flow cytometric identification of liver immune cell subsets interacting with FITC-labeled targets. Cells were first gated by forward scatter (FSC) and side scatter (SSC) to exclude debris. Live cells were discriminated using a viability dye. Subsequently, cells positive for FITC (labeled targets) were selected, followed by gating on CD45+ immune cells. Sequential gating was then performed to identify: Kupffer cells as CD11b+ , F4/80+ , and neutrophils as Ly6G+ , CD11b+ , F4/80- cells.
(TIF)

S4 Fig Intraperitoneal infection with *L. interrogans* confirms the liver as the primary organ for early leptospiral capture independent of inoculation route and sex. **(A)** Experimental schematic for intraperitoneal (i.p.) infection in male mice. Male C57BL/6 mice were i.p. injected with 10⁸ *L. interrogans* serovar Lai (strain 56601). Bacterial loads in the blood, liver, kidneys, spleen, and lungs were quantified by qPCR at 10, 30, 60, and 120 min post-infection (p.i.). **(B–E)** In each graph, the bars from left to right represent the leptospiral loads in the blood, liver, kidneys, spleen, and lungs at 10 min **(B)**, 30 min **(C)**, 60 min **(D)**, and 120 min **(E)** p.i. (n = 6). **(F)** Experimental schematic for i.p. infection in female mice. Female C57BL/6 mice were i.p. injected with 10⁸ *L. interrogans* serovar Lai (strain 56601). Bacterial loads were quantified at the indicated time points. **(G–J)** Leptospiral loads in female mice at 10 min (G), 30 min **(H)**, 60 min **(I)**, and 120 min **(J)** p.i. (n = 6). **(K)** Experimental schematic for i.p. infection in male mice followed by cardiac perfusion. Male C57BL/6 mice were i.p. injected with 10⁸ *L. interrogans*. Prior to tissue collection at the indicated time points, mice underwent rigorous cardiac perfusion with sterile PBS to eliminate blood from the tissue vasculature. **(L–O)** Leptospiral loads in perfused male mice at 10 min **(L)**, 30 min **(M)**, 60 min **(N)**, and 120 min **(O)** p.i. (n = 6). Data are presented as mean ± SEM. Statistical significance was determined with Student’s t test (unpaired, two-tailed). *p < 0.05, **p < 0.01, ***p < 0.001, ns = non-significant.
(TIF)

S5 Fig Neutralization of TNF-α does not affect early hepatic leptospiral capture. **(A)** Experimental schematic. Male C57BL/6 mice were pretreated with a neutralizing anti-TNF-α antibody (MP6-XT22) or isotype control via i.p. injection. Two hours later, mice were intravenously infected with 10⁸ *L. interrogans* serovar Lai (strain 56601). Blood and liver samples were collected at 10, 30, and 60 min post-infection (p.i.). **(B–D)** Serum TNF-α levels in control and anti-TNF-α-treated mice at 10 min **(B)**, 30 min **(C)**, and 60 min **(D)** p.i., measured by ELISA (n = 6). (**E**–**G**) Hepatic leptospiral loads in control and anti-TNF-α-treated mice at 10 min **(E)**, 30 min **(F)**, and 60 min **(G)** p.i., quantified by qPCR (n = 6). Data are presented as mean ± SEM. Statistical significance was determined with Student’s t test (unpaired, two-tailed). n.s., *p < 0.05, ***p < 0.001, ns = non-significant.
(TIF)

S6 Fig Mannan injection activates the complement system via the lectin pathway. **(A)** Experimental schematic. Male C57BL/6 mice were intravenously injected with mannan (400 μg) or vehicle control. Blood samples were collected at 2 min, 1 h, 2 h, and 4 h post-injection. **(B)** Serum complement C3 levels at 2 min, 1 h, 2 h, and 4 h post-injection, measured by ELISA (n = 6). **(C)** Serum complement C5 levels at 2 min, 1 h, 2 h, and 4 h post-injection, measured by ELISA (n = 6). **(D)** Experimental schematic. Male C57BL/6 mice were intravenously injected with mannan (400 μg) or vehicle control. Two minutes later, mice were intravenously infected with 10⁸ *L. interrogans* serovar Lai (strain 56601). Blood samples were collected at 10, 30, and 60 min post-infection (p.i.). **(E–G)** Serum complement C3 levels in control and mannan-treated mice at 10 min **(E)**, 30 min **(F)**, and 60 min **(G)** p.i., measured by ELISA (n = 6). **(H–J)** Serum complement C5 levels in control and mannan-treated mice at 10 min **(H)**, 30 min **(I)**, and 60 min **(J)** p.i., measured by ELISA (n = 6). Data are presented as mean ± SEM. Statistical significance was determined with Student’s t test (unpaired, two-tailed). **p < 0.01, ns = non-significant.
(TIF)

S1 Table Sequences of primers used for qPCR assays. List of oligonucleotide primer sequences employed for quantitative real‑time PCR (qPCR) to detect mouse target genes (Clec4d, 16s-lep, LipL32, Cebpb, and reference gene Gapdh) as well as Leptospira interrogans 16S rRNA.
(DOCX)

S2 Table Transcription factors predicted to bind the Clec4d promoter by the ChEA transcription factor targets database. The table presents candidate transcription factors (TFs) that are computationally predicted to interact with the regulatory region of the mouse Clec4d gene.
(XLSX)

S3 Table Expression levels (FPKM values) of transcription factors associated with Clec4d that are upregulated following *L. interrogans* infection. RNA‑seq‑derived FPKM (fragments per kilobase of transcript per million mapped reads) values for transcription factors (TFs) that are both predicted to regulate Clec4d (based on ChEA analysis) and significantly upregulated in Kupffer cells at 60 min post‑infection with Leptospira interrogans. Only TFs with increased expression (fold change > 2, *p* < 0.05) are shown.
(DOCX)

S1 Movie Normal liver capture of *L. interrogans* in mice. *L. interrogans* were labeled with FITC (green) and intravenously inoculated at 1 × 10^8^ for real-time imaging in mice. KCs and liver sinusoidal vasculature were stained with AF647-conjugated anti-F4/80 (red) and AF594-conjugated anti-CD31 (cyan), respectively. Quantitative analysis is presented in Fig 2I.
(MP4)

S2 Movie Reduced liver capture of *L. interrogans* in CLL-treated mice. *L. interrogans* were labeled with FITC (green) and intravenously inoculated at 1 × 10^8^ for real-time imaging in mice. KCs and liver sinusoidal vasculature were stained with AF647-conjugated anti-F4/80 (red) and AF594-conjugated anti-CD31 (cyan), respectively. Quantitative analysis is presented in Fig 3L.
(MP4)
