# Supplementary figures and images for: Leptospiral dissemination is restrained by liver macrophages through Clec4d-driven capture via C/EBPβ activation
Source: PLoS Pathog. 2026 May 13;22(5):e1014232. doi: 10.1371/journal.ppat.1014232 (PMC13189408; doi:10.1371/journal.ppat.1014232)

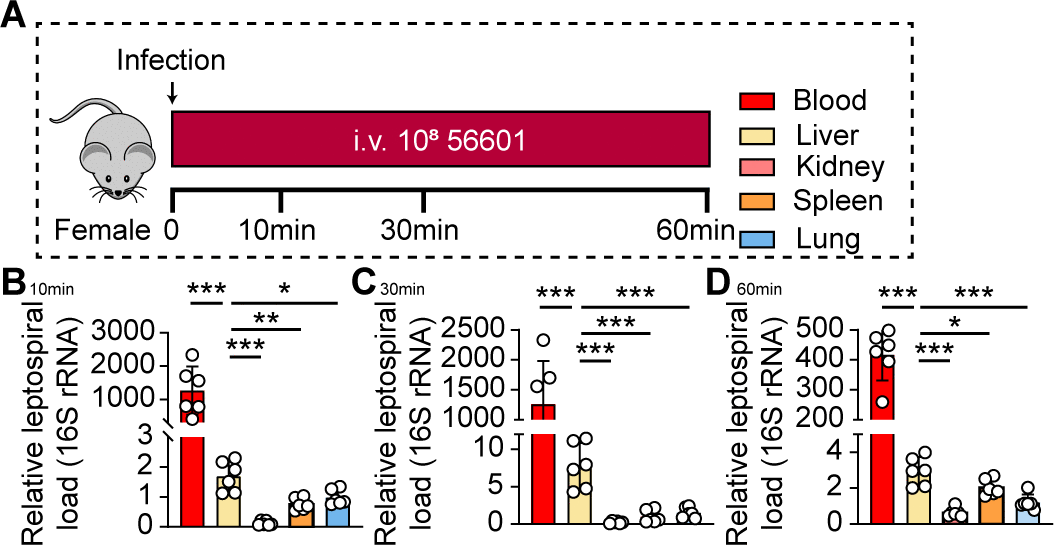

Supplement: S1 Fig — (A) Experimental schematic for determining bacterial distribution in female mice. Female C57BL/6 mice were intravenously infected with 10⁸ L. interrogans serovar Lai (strain 56601). Bacterial loads in the liver, kidneys, spleen, lungs, and blood were quantified by qPCR at 10, 30, and 60 minutes post-infection (p.i.). (B-D) In each graph, the bars from left to right represent the leptospiral loads in the blood, liver, kidneys, spleen, and lungs at 10 min (B), 30 min (C), and 60 min (D) p.i. (n = 6). Data are presented as mean ± SEM. Statistical significance was determined with Student’s t test (unpaired, two-tailed). *p < 0.05, **p < 0.01, ***p < 0.001. (TIF) [file ppat.1014232.s001.tif]

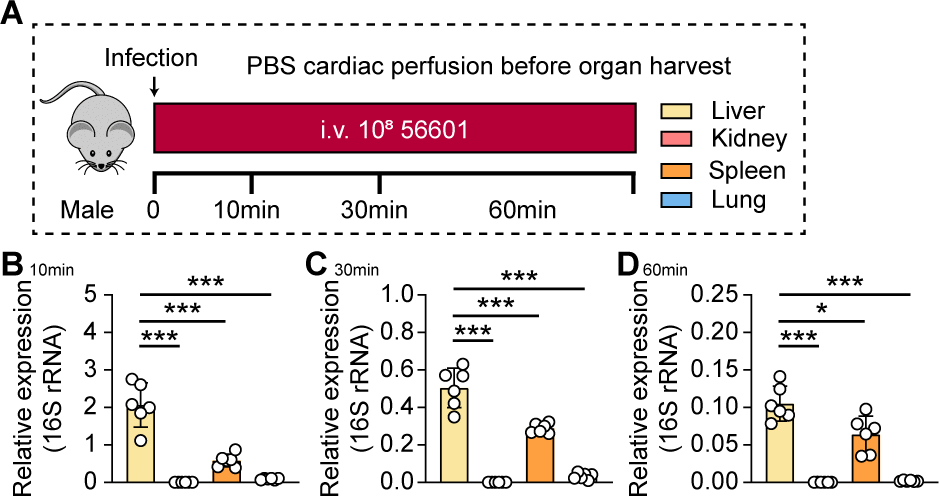

Supplement: S2 Fig — (A) Experimental schematic for determining bacterial distribution after cardiac perfusion in male mice. C57BL/6 mice were intravenously infected with 10⁸ L. interrogans serovar Lai (strain 56601). Prior to tissue collection at 10, 30, and 60 minutes p.i., mice underwent rigorous cardiac perfusion with sterile PBS to eliminate blood from the tissue vasculature. (B-D) In each graph, the bars from left to right represent the leptospiral loads in the liver, kidneys, spleen, and lungs of perfused male mice at 10 min (B), 30 min (C), and 60 min (D) p.i. (n = 6). Data are presented as mean ± SEM. Statistical significance was determined with Student’s t test (unpaired, two-tailed). *p < 0.05, ***p < 0.001. (TIF) [file ppat.1014232.s002.tif]

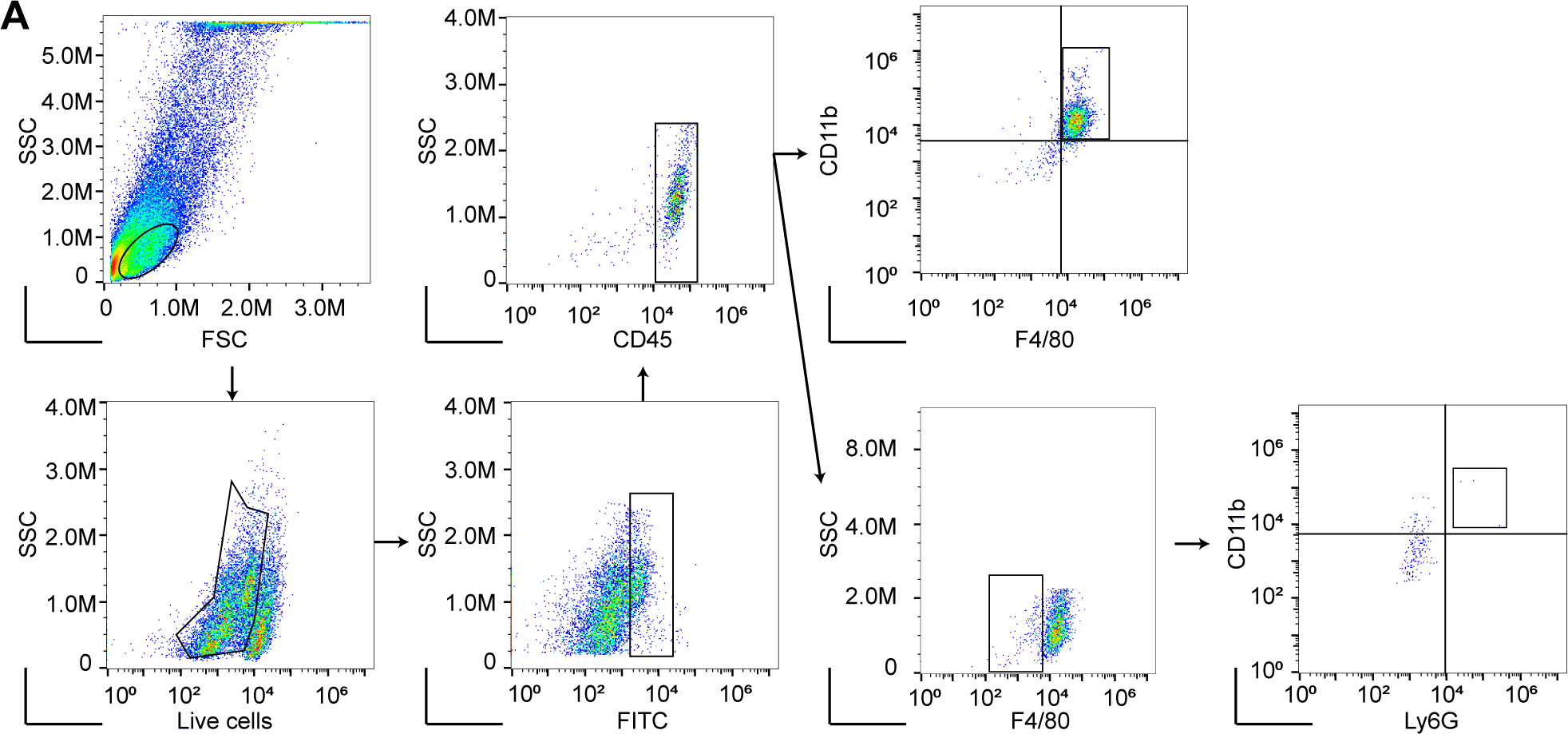

Supplement: S3 Fig — (A) Gating strategy for flow cytometric identification of liver immune cell subsets interacting with FITC-labeled targets. Cells were first gated by forward scatter (FSC) and side scatter (SSC) to exclude debris. Live cells were discriminated using a viability dye. Subsequently, cells positive for FITC (labeled targets) were selected, followed by gating on CD45+ immune cells. Sequential gating was then performed to identify: Kupffer cells as CD11b+ , F4/80+ , and neutrophils as Ly6G+ , CD11b+ , F4/80- cells. (TIF) [file ppat.1014232.s003.tif]

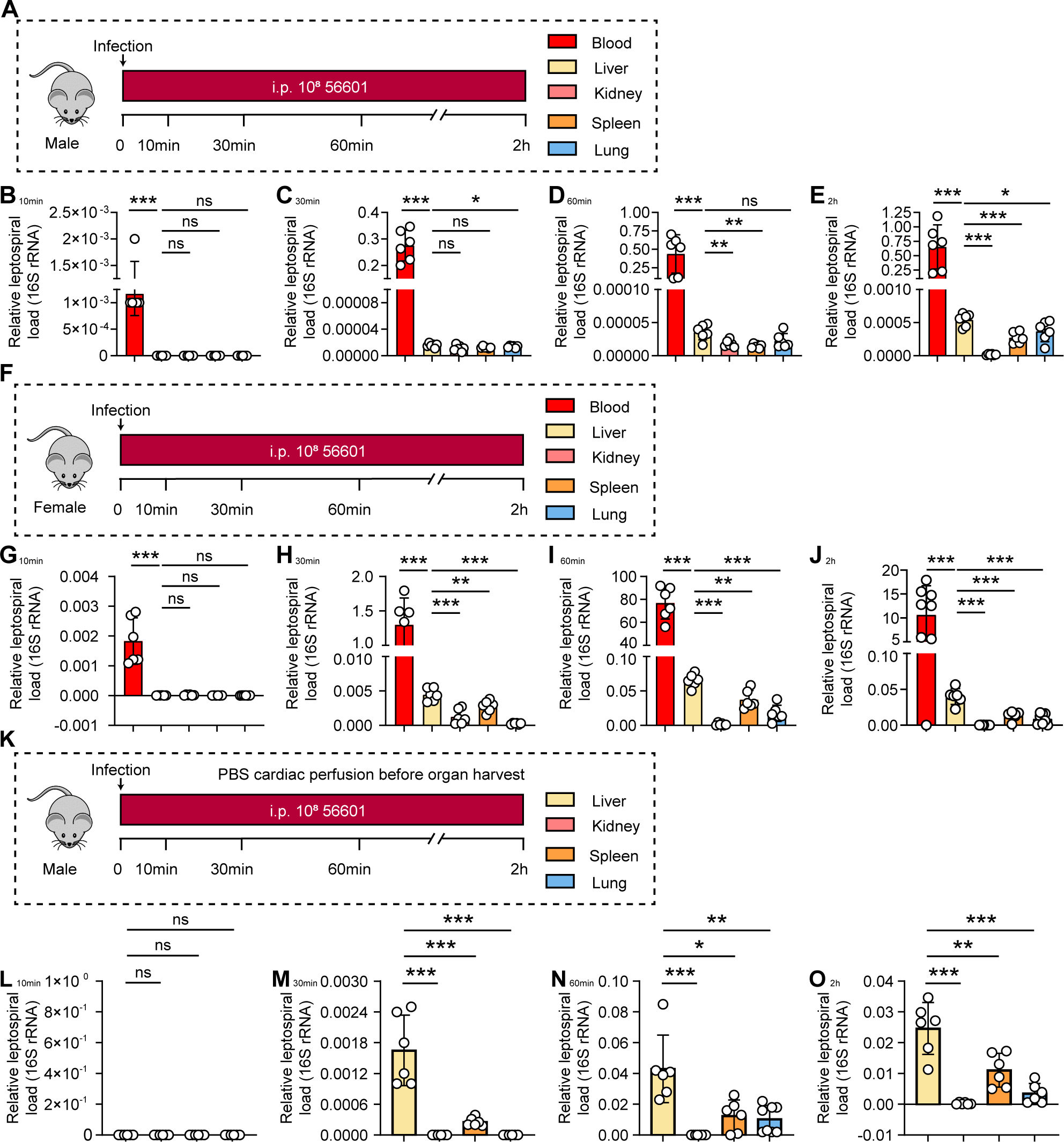

Supplement: S4 Fig — (A) Experimental schematic for intraperitoneal (i.p.) infection in male mice. Male C57BL/6 mice were i.p. injected with 10⁸ L. interrogans serovar Lai (strain 56601). Bacterial loads in the blood, liver, kidneys, spleen, and lungs were quantified by qPCR at 10, 30, 60, and 120 min post-infection (p.i.). (B–E) In each graph, the bars from left to right represent the leptospiral loads in the blood, liver, kidneys, spleen, and lungs at 10 min (B), 30 min (C), 60 min (D), and 120 min (E) p.i. (n = 6). (F) Experimental schematic for i.p. infection in female mice. Female C57BL/6 mice were i.p. injected with 10⁸ L. interrogans serovar Lai (strain 56601). Bacterial loads were quantified at the indicated time points. (G–J) Leptospiral loads in female mice at 10 min (G), 30 min (H), 60 min (I), and 120 min (J) p.i. (n = 6). (K) Experimental schematic for i.p. infection in male mice followed by cardiac perfusion. Male C57BL/6 mice were i.p. injected with 10⁸ L. interrogans. Prior to tissue collection at the indicated time points, mice underwent rigorous cardiac perfusion with sterile PBS to eliminate blood from the tissue vasculature. (L–O) Leptospiral loads in perfused male mice at 10 min (L), 30 min (M), 60 min (N), and 120 min (O) p.i. (n = 6). Data are presented as mean ± SEM. Statistical significance was determined with Student’s t test (unpaired, two-tailed). *p < 0.05, **p < 0.01, ***p < 0.001, ns = non-significant. (TIF) [file ppat.1014232.s004.tif]

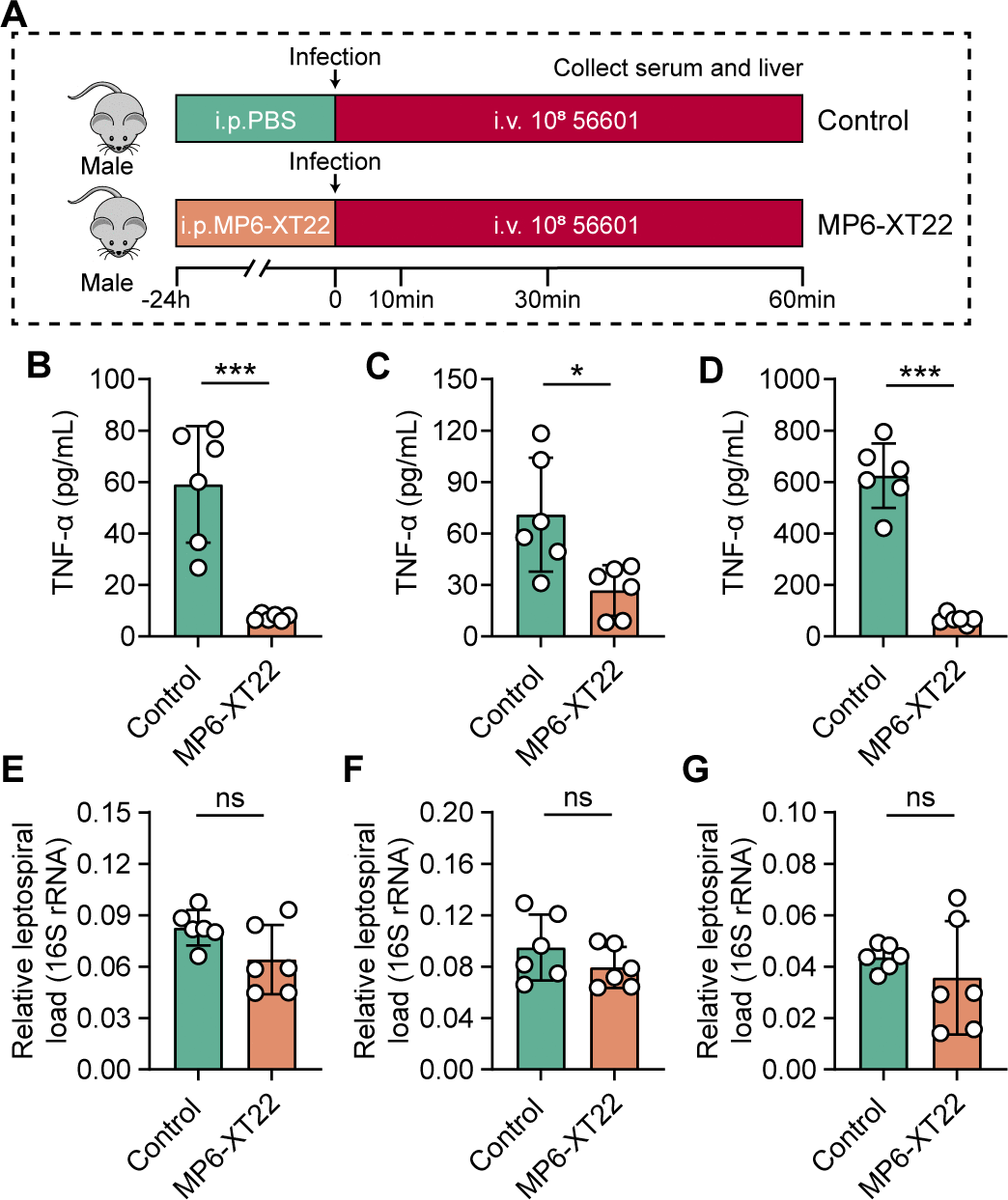

Supplement: S5 Fig — (A) Experimental schematic. Male C57BL/6 mice were pretreated with a neutralizing anti-TNF-α antibody (MP6-XT22) or isotype control via i.p. injection. Two hours later, mice were intravenously infected with 10⁸ L. interrogans serovar Lai (strain 56601). Blood and liver samples were collected at 10, 30, and 60 min post-infection (p.i.). (B–D) Serum TNF-α levels in control and anti-TNF-α-treated mice at 10 min (B), 30 min (C), and 60 min (D) p.i., measured by ELISA (n = 6). (E–G) Hepatic leptospiral loads in control and anti-TNF-α-treated mice at 10 min (E), 30 min (F), and 60 min (G) p.i., quantified by qPCR (n = 6). Data are presented as mean ± SEM. Statistical significance was determined with Student’s t test (unpaired, two-tailed). n.s., *p < 0.05, ***p < 0.001, ns = non-significant. (TIF) [file ppat.1014232.s005.tif]

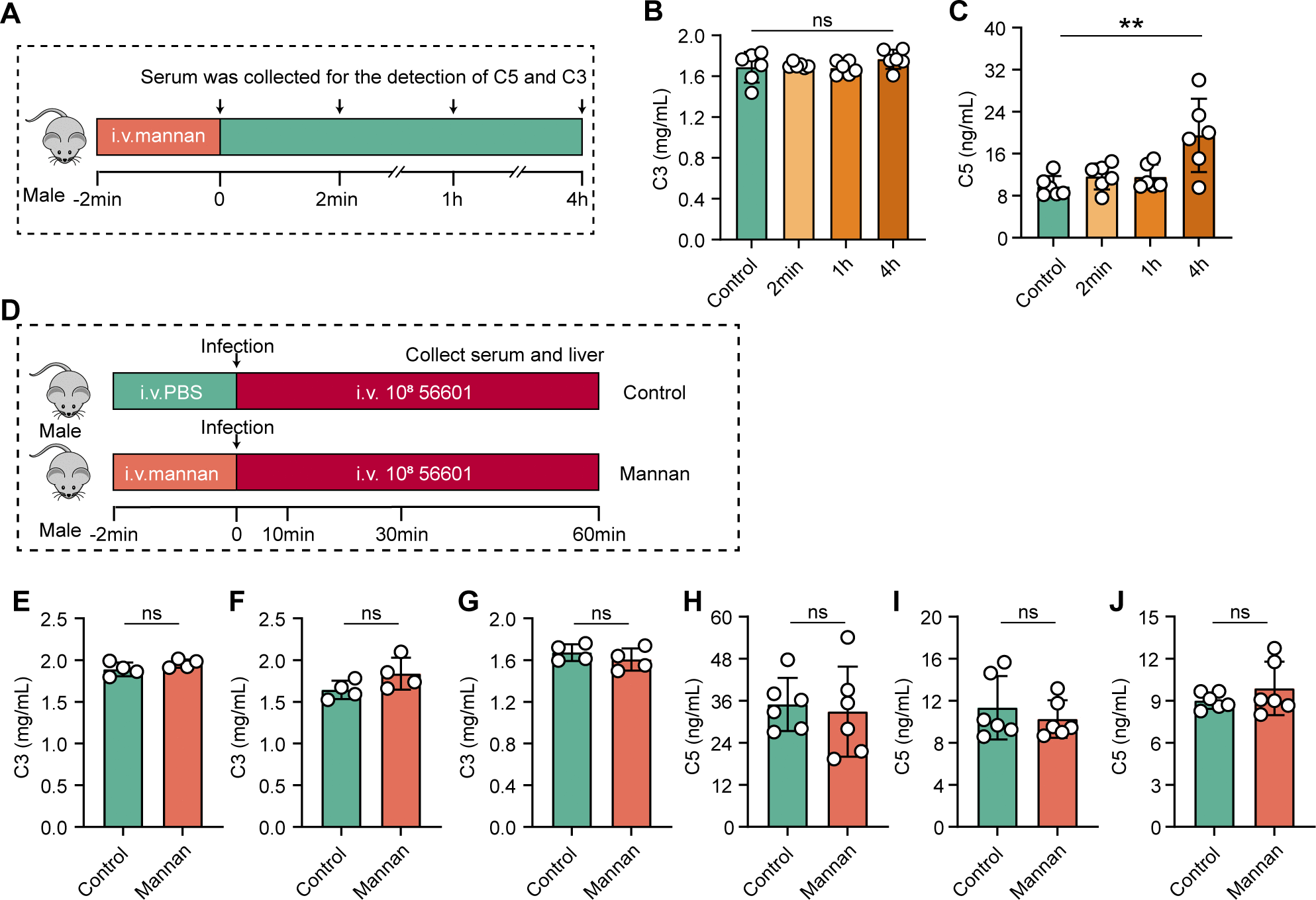

Supplement: S6 Fig — (A) Experimental schematic. Male C57BL/6 mice were intravenously injected with mannan (400 μg) or vehicle control. Blood samples were collected at 2 min, 1 h, 2 h, and 4 h post-injection. (B) Serum complement C3 levels at 2 min, 1 h, 2 h, and 4 h post-injection, measured by ELISA (n = 6). (C) Serum complement C5 levels at 2 min, 1 h, 2 h, and 4 h post-injection, measured by ELISA (n = 6). (D) Experimental schematic. Male C57BL/6 mice were intravenously injected with mannan (400 μg) or vehicle control. Two minutes later, mice were intravenously infected with 10⁸ L. interrogans serovar Lai (strain 56601). Blood samples were collected at 10, 30, and 60 min post-infection (p.i.). (E–G) Serum complement C3 levels in control and mannan-treated mice at 10 min (E), 30 min (F), and 60 min (G) p.i., measured by ELISA (n = 6). (H–J) Serum complement C5 levels in control and mannan-treated mice at 10 min (H), 30 min (I), and 60 min (J) p.i., measured by ELISA (n = 6). Data are presented as mean ± SEM. Statistical significance was determined with Student’s t test (unpaired, two-tailed). **p < 0.01, ns = non-significant. (TIF) [file ppat.1014232.s006.tif]
