# Supplementary material for: Leptospiral dissemination is restrained by liver macrophages through Clec4d-driven capture via C/EBPβ activation
Source: PLoS Pathog. 2026 May 13;22(5):e1014232. doi: 10.1371/journal.ppat.1014232 (PMC13189408; doi:10.1371/journal.ppat.1014232)
Supplement: S1 Table — List of oligonucleotide primer sequences employed for quantitative real‑time PCR (qPCR) to detect mouse target genes (Clec4d, 16s-lep, LipL32, Cebpb, and reference gene Gapdh) as well as Leptospira interrogans 16S rRNA. (DOCX) [file ppat.1014232.s007.docx]

**Table 1: Sequence of primers used for qPCR assays.**

| **Primer name** | **Primer sequence (5’ to 3’)** |
| --- | --- |
| GAPDH Sense | AGGTCGGTGTGAACGGATTTG |
| GAPDH Antisense | GGGGTCGTTGATGGCAACA |
| Cebpb Sense | CAAGAAGACGGTGGACAAGC |
| Cebpb Antisense | AGCTGCTCCACCTTCTTCTG |
| Clec4d Sense | GGAAAGTCATTCCAGACCCA |
| Clec4d Antisense | AAGACGCCATTTAACCCACA |
| 16s-lep Sense | AGCACGTGTGTTGCCCTAGACATA |
| 16s-lep Antisense | GTTGCCATCATTCAGTTGGGCACT |
| LipL32 Sense | TCGCTGAAATRGGWGTTCGT |
| LipL32 Antisense | CGCCTGGYTCMCCGATT |
