# Supplementary material for: Leptospiral dissemination is restrained by liver macrophages through Clec4d-driven capture via C/EBPβ activation
Source: PLoS Pathog. 2026 May 13;22(5):e1014232. doi: 10.1371/journal.ppat.1014232 (PMC13189408; doi:10.1371/journal.ppat.1014232)
Supplement: S3 Table — RNA‑seq‑derived FPKM (fragments per kilobase of transcript per million mapped reads) values for transcription factors (TFs) that are both predicted to regulate Clec4d (based on ChEA analysis) and significantly upregulated in Kupffer cells at 60 min post‑infection with Leptospira interrogans. Only TFs with increased expression (fold change > 2, p < 0.05) are shown. (DOCX) [file ppat.1014232.s009.docx]

**Table 3: The FPKM value of transcription factors (TFs) associated with *Clec4d* with upregulated expression after *L. interrogans*** **infection.**

| Gene name | CON1 | CON2 | CON3 | IF1 | IF2 | IF3 |
| --- | --- | --- | --- | --- | --- | --- |
| RUNX1 | 1.82 | 1.26 | 1.63 | 4.88 | 4.85 | 3.71 |
| GATA2 | 0.36 | 0.37 | 0.41 | 0.88 | 0.89 | 2.82 |
| GFI1B | 0.03 | 0 | 0 | 0.27 | 0.05 | 0 |
| TAL1 | 0.04 | 0.10 | 0.14 | 0.15 | 0.27 | 0.09 |
| CEBPB | 359.54 | 271.59 | 390.02 | 586.94 | 544.83 | 532.13 |
